# Transforming growth factor-β2 is associated with atherosclerotic plaque stability and lower risk for cardiovascular events

**DOI:** 10.1093/cvr/cvad079

**Published:** 2023-05-18

**Authors:** Andreas Edsfeldt, Pratibha Singh, Frank Matthes, Christoffer Tengryd, Michele Cavalera, Eva Bengtsson, Pontus Dunér, Petr Volkov, Glykeria Karadimou, Anton Gisterå, Marju Orho-Melander, Jan Nilsson, Jiangming Sun, Isabel Gonçalves

**Affiliations:** Department of Clinical Sciences, Malmö, Lund University, Lund, Sweden; Wallenberg Centre for Molecular Medicine, Lund University, Lund, Sweden; Department of Cardiology, Skåne University Hospital, Malmö, Sweden; Department of Clinical Sciences, Malmö, Lund University, Lund, Sweden; Department of Clinical Sciences, Malmö, Lund University, Lund, Sweden; Department of Clinical Sciences, Malmö, Lund University, Lund, Sweden; Department of Clinical Sciences, Malmö, Lund University, Lund, Sweden; Department of Clinical Sciences, Malmö, Lund University, Lund, Sweden; Faculty of Health and Society, Malmö University, Malmö, Sweden; Biofilms—Research Center for Biointerfaces, Malmö University, Malmö, Sweden; Department of Clinical Sciences, Malmö, Lund University, Lund, Sweden; Department of Clinical Sciences, LUDC Bioinformatics Unit, Malmö, Lund University, Lund, Sweden; Data Science and Quantitative Biology, Discovery Sciences, R&D, AstraZeneca, Gothenburg, Sweden; Department of Molecular Medicine and Surgery, Karolinska Institute, Stockholm, Sweden; Department of Medicine, Center for Molecular Medicine, Solna, Karolinska University Hospital, Karolinska Institutet, Stockholm, Sweden; Department of Clinical Sciences, Malmö, Lund University, Lund, Sweden; Department of Clinical Sciences, Malmö, Lund University, Lund, Sweden; Department of Clinical Sciences, Malmö, Lund University, Lund, Sweden; Department of Clinical Sciences, Malmö, Lund University, Lund, Sweden; Department of Cardiology, Skåne University Hospital, Malmö, Sweden

**Keywords:** Atherosclerosis, Plaque stability, Extracellular matrix, TGF-β, Inflammation

## Abstract

**Aims:**

Transforming growth factor-beta (TGF-β) exists in three isoforms TGF-β1, -β2, and -β3. TGF-β1 has been suggested to be important for maintaining plaque stability, yet the role of TGF-β2 and -β3 in atherosclerosis remains to be investigated.

This study explores the association of the three isoforms of TGF-β with plaque stability in the human atherosclerotic disease.

**Methods and results:**

TGF-β1, -β2, and -β3 proteins were quantified in 223 human carotid plaques by immunoassays. Indications for the endarterectomy were: symptomatic carotid plaque with stenosis >70% or without symptoms and >80% stenosis. Plaque mRNA levels were assessed by RNA sequencing. Plaque components and extracellular matrix were measured histologically and biochemically. Matrix metalloproteinases and monocyte chemoattractant protein-1 (MCP-1) was measured with immunoassays. The effect of TGF-β2 on inflammation and protease activity was investigated *in vitro* using THP-1 and RAW264.7 macrophages. Patients were followed longitudinally for cardiovascular (CV) events.

TGF-β2 was the most abundant isoform and was increased at both protein and mRNA levels in asymptomatic plaques. TGF-β2 was the main determinant separating asymptomatic plaques in an Orthogonal Projections to Latent Structures Discriminant Analysis. TGF-β2 correlated positively to features of plaque stability and inversely to markers of plaque vulnerability. TGF-β2 was the only isoform inversely correlated to the matrix-degrading matrix metalloproteinase-9 and inflammation in the plaque tissue. *In vitro*, TGF-β2 pre-treatment reduced MCP-1 gene and protein levels as well as matrix metalloproteinase-9 gene levels and activity. Patients with plaques with high TGF-β2 levels had a lower risk to suffer from future CV events.

**Conclusions:**

TGF-β2 is the most abundant TGF-β isoform in human plaques and may maintain plaque stability by decreasing inflammation and matrix degradation.

## Graphical Abstract

**Graphical Abstract:**
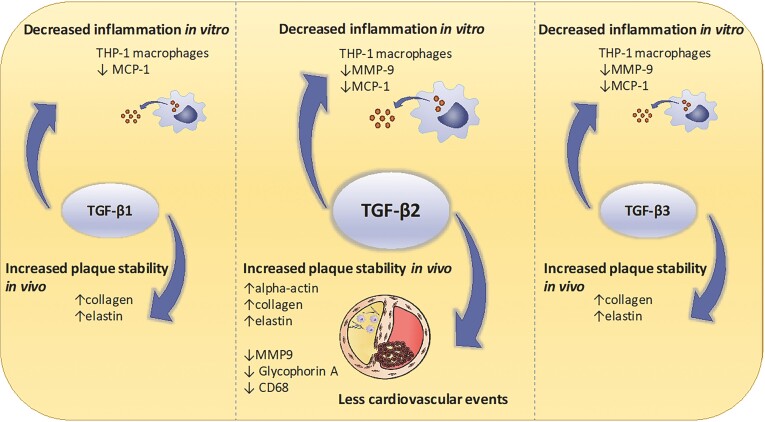
TGF-β2 decreases inflammation and is associated with factors important for plaque stability.

**Time of primary review: 41 days**

## 1. Introduction

Atherosclerosis is a chronic systemic disease of the large and medium-sized arteries as a result of lipoprotein retention in the intima with ensuing inflammatory and reparative responses.^1^ The balance between these two types of responses is essential for the phenotype of the atherosclerotic plaques. Stable plaques have thick fibrous caps with less inflammation and lipids, whereas the high-risk or rupture-prone plaques have thin caps, reduced extracellular matrix (ECM) are inflamed and lipid-rich.

Even though improved therapies have led to a decrease in CV mortality, stroke and myocardial infarction (MI) commonly caused by atherosclerotic plaque ruptures or erosions, remain the most common causes of morbidity and mortality globally. Therefore, discovering new pathways of vascular repair and plaque stability would be of great importance for developing new therapeutic strategies.^2^ One such pathway could be the transforming growth factor-beta (TGF-β) signaling pathway that has been suggested to be anti-atherogenic.^3–7^ The TGF-β isoforms (-β1, -β2, and -β3) are cytokines/growth factors of the TGF-β signaling family, each encoded by unique genes.^8,9^ The TGF-βs regulate cellular proliferation/differentiation, tissue repair, ECM production, and inflammatory responses.^3,4,10–12^

Until now, most of the *in vitro* and *in vivo* studies on the role of TGF-β in atherosclerosis have focused on TGF-β1 alone, on neutralization of all three isoforms simultaneously, or on manipulation of the TGF-β signaling pathway.^5,6^ Importantly, functional studies of TGF-β2 and TGF-β3 in atherosclerosis are currently lacking.

The present study aimed to identify the presence of TGF-β isoforms that are present in human atherosclerotic plaques and if specific isoforms are associated with a particular plaque phenotype, that is stable or rupture-prone. Additionally, we explored possible underlying plaque stabilizing mechanisms, using *in vitro* experiments. Finally, we aimed to identify if any of the isoforms could predict future CV events.

## 2. Methods

*A detailed methods description is provided in the* supplementary material.

### 2.1 Study design

Two hundred-twenty-three human carotid plaques were obtained from 218 patients who underwent carotid endarterectomy (CEA) between 2005 and 2011 at the Vascular Department at Skåne University Hospital (Malmö, Sweden). Five patients underwent surgery for bilateral carotid plaques. All the patients gave written and oral informed consent. The study was approved by the Regional Ethical Committee and conformed to the principles of the Declaration of Helsinki. Clinical characteristics of the patients are described in Supplementary material online, *Table S1*. Patients were considered symptomatic if they suffered from amaurosis fugax (AF), transient ischemic attack (TIA), or ischemic stroke within one month prior to surgery. The indications for CEA were (i) ipsilateral symptoms and stenosis >70% or (ii) asymptomatic plaque with >80% stenosis as assessed by duplex ultrasound, as previously described.^13^ No power calculation was possible due to the design of the study. The inclusion was done consecutively and no exclusion criteria were used to avoid selection bias (except the ability to provide informed consent). The clinical data in this study was collected by research nurses at the time of inclusion. The *in vitro* experiments consist of both analyses of levels of proteins and mRNA in plaque tissue, as well as cell culture experiments with THP-1 macrophages. The data were processed randomly and blinded for the technical staff.

### 2.2 Sample preparation

Plaques were snap-frozen directly after surgical removal and weighed on dry ice. From the most stenotic part of the plaque, a 1 mm thick section was taken for histology and embedded in optimal cutting temperature compound medium. An adjacent section of 1 mm was kept for RNA sequencing. The rest of the plaque was homogenized in a standardized way, described earlier.^14^

### 2.3 Histology and immunohistochemistry

The most stenotic part of the plaque (1 mm) was sectioned for histology as previously described.^1^ Sections were fixed with Histochoice (Amresco, Solon, OH, USA) and stained for smooth muscle cells (smooth muscle alpha-actin), macrophages (CD68), neutral lipids (Oil Red O), intraplaque hemorrhage (Glycophorin A), and collagen (Movat pentachrome) as previously described.^14–16^ The histological vulnerability index (VI) was calculated as a ratio between plaque areas of stabilizing components (collagen, smooth muscle alpha-actin) and components associated to vulnerability (lipids, macrophages, and intra-plaque haemorrhage) as previously described.^16^ Calcified areas in plaques were evaluated as described previously.^17^ To stain for the three TGF-β isoforms the frozen tissue was fixated in 4% buffered formaldehyde solution (Histolab Products AB) overnight. The tissue was then dehydrated in increasing alcohol concentrations, cleared in Xylen and embedded in Histowax (Histolab Products AB). Mouse monoclonal antibodies were used to stain for TGF-β1 (Abcam, Cambridge, UK, ab27969, 0.06 µg/mL) and TGF-β2 (Abcam, Cambridge, UK, ab36495, 0.25 µg/mL) overnight at 4°C. A mouse monoclonal isotype control antibody was used as a control (Abcam, Cambridge, UK, ab81032, at corresponding concentration). A rabbit polyclonal antibody was used to stain for TGF-β3 (Abcam, Cambridge, UK, ab15537; 0293 mg/mL) together with a rabbit polyclonal isotype control antibody (Abcam, Cambridge, UK, ab37415, 5 mg/mL). To detect staining a MACH3 probe and horseradish peroxidase polymer (Biocare Medical, Pacheco, CA, USA) was used.

Positive immunoreactivity was visualized using 3,3′ and immunoreactivity was quantified using the imaging software program BioPix iQ version 2.3.1 (Biopix Ab, Gothenburg, Sweden).

### 2.4 Plaque TGF-β1, -β2, and -β3 analysis

TGF-β1, -β2, and -β3 were assessed in 25 µL of supernatant from plaque homogenate (similar to cell culture supernatants) after centrifugation for 5 min at 6220 RCF in 4°C, using the Milliplex Map TGF-β Magnetic Bead 3 Plex Kit—Immunology Multiplex Assay from MerckMillipore (TGFBMAG-64K-03, Billerica, MA, USA) according to the manufacturer’s instructions and measured using Luminex 100 IS 2.3 (Austin, TX, USA). The levels of TGF-β1, -β2, and -β3 were normalized to plaque wet weight to ensure normalization against all plaque components acting as reservoir for growth factors, including cells, matrix, and calcium.^18,19^ Samples were not acidified in order to measure only free TGF-β1, -β2, and -β3.

### 2.5 TGF-β plaque homogenate spiking

To confirm the accuracy of the method applied on plaque tissue homogenates, TGF-β recovery from spiked plaque homogenates was assessed using the Milliplex MAP TGF-β1/2/3 magnetic bead kit (EMD Millipore/Merck, Darmstadt, Germany). Aliquots of one plaque homogenate or assay buffer were spiked with 200 or 2000 pg/mL TGFβ-1, -2, and -3 standards from the kit. TGFβ-1, -2, and -3 concentrations were quantified in duplicates according to the manufacturer’s instructions, and recovery percentages were calculated normalizing to the respective spiked assay buffer sample as 100%.

### 2.6 Biochemical assessments of plaque glycosaminoglycans, collagen and elastin levels

The ECM components glycosaminoglycans (GAG), collagen, and elastin were analyzed in plaque homogenate with colometric assays as previously described.^14^ Values were normalized to plaque wet weight.

### 2.7 Plaque levels of cytokines, matrix metalloproteinases, and tissue inhibitors of matrix metalloproteinases

Cytokines were measured in plaque homogenate supernatants using Luminex (Millipore Corporation, MA, USA) or a proximity extension assay (Olink, Uppsala), as previously described.^15,20,21^ All cytokines were normalized to plaque wet weight. The proximity extension assay analysis was performed by a pre-processing normalization procedure using Olink Wizard for GenEx (Multid Analyses, Sweden) and all presented as arbitrary units.

Plaque homogenate levels of matrix metalloproteinases (MMPs)-1, -2, -3, -9, -10 tissue inhibitors of MMPs (TIMPs 1 and 2) were analyzed with Mesocale human MMP ultra-sensitive kit (Mesoscale, Gaithersburg, MD, USA) and MILLIPLEX MAP Human TIMP Magnetic Bead Panel (Milliplex, MA, USA) as previously described.^20^ All analyses were performed according to the manufacturer's instructions and results were normalized to plaque wet weight.

To ensure the reproducibility of the different techniques used to measure cytokines and MMPs, correlation analyses for four overlapping markers [IL-6, monocyte chemoattractant protein-1 (MCP-1), MMP-1 and MMP-10] were performed. Importantly, strong positive correlations were identified for all markers (correlation coefficient range 0.71–0.85, *P*-value range 2.5 × 10^−30^–4.6 × 10^−54^).

### 2.8 LPS stimulation of THP-1 cells

Human THP-1 blood monocytes were treated with Phorbol 12-Myristate 13-Acetate (PMA, 100 ng/mL) for 24 h, followed by 6 days of resting. The differentiated cells were treated with 5 ng/mL TGF-β1, -β2, or -β3 for 48 h and successively with lipopolysaccharide (LPS) for 15 h to polarise towards a pro-inflammatory macrophage phenotype.

### 2.9 MCP-1 release *in vitro*

To assess the effect of TGF-β treatment on MCP-1 protein release PMA-differentiated THP-1 cells (100 ng/mL PMA for 24 h, followed by 6 days resting phase) were stimulated with the three different TGF-β isoforms (5 ng/mL for 48 h) prior to LPS stimulation (100 ng/mL for 15 h). Human MCP-1 was measured in cell culture supernatants using ELISA MAX kits (BioLegend, London, UK) according to the manufacturer’s instructions.

### 2.10 MMP-9 activity *in vitro*

THP-1 cells were differentiated using PMA (24 h, 100 ng/mL, followed by 6 days resting phase), treated with TGF-β (48 h, 10 ng/mL) and stimulated with 1 µg/mL LPS (24 h). MMP-9 activity in the supernatant was measured using the Fluorokine E Human Active MMP-9 Fluorescent Assay kit (R&D Systems, Abingdon, UK) including 4-aminophenylmercuric acetate treatment according to the manufacturer’s instructions.

### 2.11 *In vitro* RAW 264.7 systems

RAW 264.7 cells were treated with TGF-β isoforms (48 h, 5 ng/mL) and stimulated with LPS (15 h, 100 ng/mL).

### 2.12 *In vitro* RNA analyses

Total RNA was extracted using the Trizol method and reverse transcribed using the High capacity RNA-to-cDNA kit (Thermo Fisher, Göteborg, Sweden). Gene expression was analyzed by quantitative real-time PCR on a QuantStudio 7 Flex instrument (Applied Biosystems/Thermo Fisher) using Taqman Fast Advanced master mix and appropriate Taqman probes. Relative gene expression was calculated with QuantStudio Software v1.1 (Thermo Fisher) using the ΔΔCt method and normalization to GAPDH expression as endogenous control. The qPCR gene expression data were normalized to the expression of endogenous control and for comparison of groups, gene expression levels are expressed as fold change expression compared to controls.

### 2.13 Human carotid plaque RNA sequencing

Expression of the TGF-β isoforms and their receptor genes were evaluated from global transcriptome RNAseq data. RNA was prepared from standard total RNA extraction with Trizol cleared of Ribosomal RNA using Ribo-Zero^™^ Magnetic Kit from (Epicentre). Strand-specific RNAseq libraries were prepared with ScriptSeq^™^ v2 RNA-Seq Library v2 Preparation Kit (Epicentre). Paired-end sequencing libraries were generated and sequenced using high-output kit version 2, HiSeq2000 platform, Illumina, USA. Finally, 60 plaques were used for differential gene expression analysis, and for Pearson correlation analysis between genes. A linear model was conducted to examine differentially expressed genes comparing patients with (Symptomatic) or without (Asymptomatic) symptomatic carotid disease. *P*-values were adjusted using the Benjamini & Hochberg method.

### 2.14 Micro-array plaque analysis in the BiKE cohort

For the microarray analysis, carotid plaques from the Biobank of Karolinska carotid Endarterectomies (BiKE; Karolinska Institute, Stockholm, Sweden) project were obtained with participants' informed written consent, per the Declaration of Helsinki and approved by the Ethical Committee of Stockholm. A total of 127 atherosclerotic plaque tissue samples were collected from patients who underwent CEA at Karolinska University Hospital, Stockholm, Sweden. After being rinsed, the plaques were frozen instantly on dry ice for subsequent isolation of RNA. Total RNA was extracted with an RNeasy Mini kit according to the instructions of the manufacturer. The tissue samples were assessed and samples with low-quality RNA and low concentrations were excluded from the study. RNA samples were hybridized and scanned at the Karolinska Institute Affymetrix core facility (Karolinska Institute) using Affymetrix HG-U133 plus 2.0 arrays. Further information is described in a previous study.^22^

### 2.15 Follow up

The primary outcome of CV events was studied longitudinally and included MI, TIAs, AF, and vascular interventions not planned at the time of the operation such as CEA, carotid artery stenting, coronary artery bypass grafting, or percutaneous coronary artery intervention as well as all deaths with an underlying CV cause. CV events were identified through the Swedish National Patient Register for all hospital discharge codes for the patients included in the study. Patients were identified through their personal identity number. The Swedish National patient register has high coverage with 99% of all somatic (including surgery) and psychiatric hospital discharges registered.^23^ The following ICD-10 codes were used to identify CV events:

- Cerebrovascular: G45.3, G45.9, G46, I63.1–5, I63.8–9, and I64.
- CV: I21–22, I24.8–9, I25.1–2, I25.5–6, I25.8.

The following ICD-10 codes from the Swedish cause of death register were used to identify CV deaths.^24^:

- Cerebrovascular: I63.2, I64, I69.4, I73.9, I74.9, and I99.
- CV: I20,9, I21.9, I25.1, I25.5, I25.8–9, I46.9.

All events were verified by patient medical charts and telephone interviews. Registered events are presented in group form in Supplementary material online, *Table S2*. Events occurring within 72 h after CEA were considered procedure-related and were not included in the analysis. For patients suffering from multiple events, only the first was taken into account in the survival analysis.

### 2.16 Statistical methods

Non-normally distributed continuous variables were presented as median with inter quartile range (IQR), while categorical variables are expressed as percentages. For continuous variables, the Mann–Whitney *U* test and Spearman's rank correlation were used and for categorical variables, the Chi-square test was performed.

Kaplan–Meier survival analyses were performed for the highest quartile vs. the lower three quartiles of each TGF-β isoform. Significant differences between the groups were assessed by the Log-rank test. Cox proportional hazard regression analysis [hazard ratios (HR) with 95% CI] was used to analyze the time to event association between baseline characteristics and risk of CV events [hazard ratios (HR) with 95% CI]. Multivariable models were performed using known CV risk as covariates: age, sex, hypertension, LDL, HDL, and current smoking. A *P*-value of <0.05 was considered statistically significant. Statistical analysis was performed using SPSS 24.0 (IBM Corp., Amonk, NY, USA).

*P*-values potentially affected by multiple comparison were adjusted using the two-stage step-up method of Benjamin, Krieger, and Yekutieli with a false discovery rate <5% [GraphPad Prism (v9.0.2)] and are reported as *q*-values.

For RNA sequencing analysis, Surrogate Variable Analysis using svaseq^25^ identified four significant surrogate variables. Next, fold changes between symptomatic and asymptomatic patients were estimated using DESeq2^26^ with the surrogate variables as covariates and genes were considered differentially expressed if the Wald test *P*-value was <0.05.

Principal component analysis (PCA) and orthogonal partial least squares discriminant analysis (OPLS-DA) were carried out in SIMCA-P software package (version 14.1 and 15, Umetrics, Umeå, Sweden) using log-transformed, mean-centered, and scaled to unified variance input variables. The overall contribution of each variable to group discrimination was ranked by variable influence on projection (VIP) values. In brief, VIP scores are calculated from a weighted sum of the squared correlations of model components with the original variables. The weights represent the percentage variance explained by the model components. The highest score is assigned to the most contributory variable in class discrimination. Variables with a VIP value of >1 were considered relevant to group discrimination. Ellipse based on Hotelling’s T2 represents the 95% confidence interval of modeled variation in score plots.

The discriminatory power of selected variables (VIP > 1) to cluster into two groups was further examined by *K*-means unsupervised clustering in Qlucore omics explorer v 3.5. Hierarchichal clustering of variables was performed using the Elucidean distance and average linkage method. Dataset was log-transformed, mean-centered, and normalized, to unit variance for analysis in Qlucore omics explorer v 3.5 and presented as a heatmap.

## 3. Results

### 3.1 TGF-β2 is the most abundant TGF-β isoform in human carotid plaques

**Figure 1:**
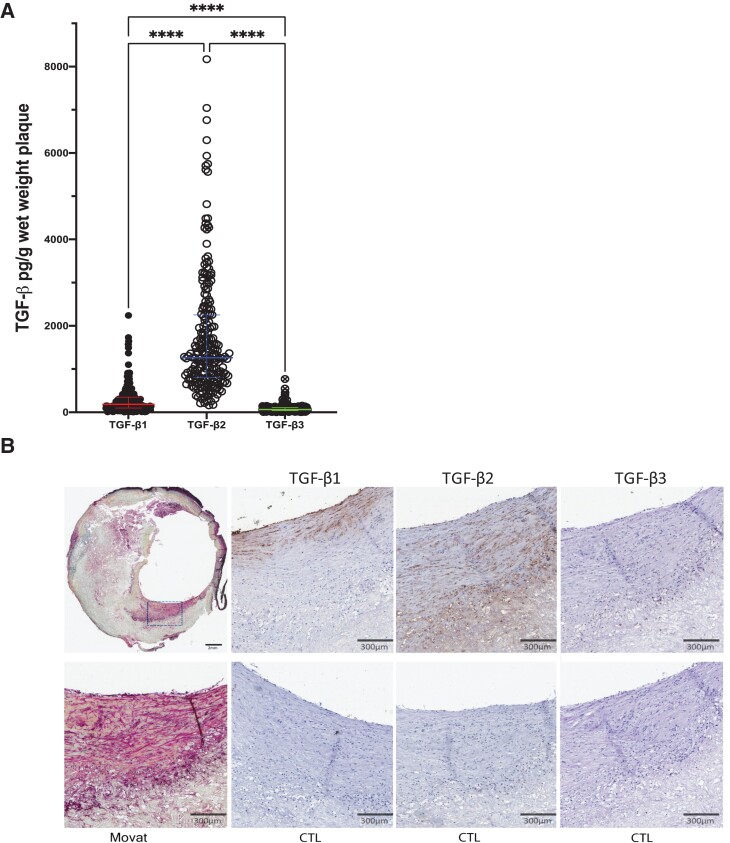
TGF-β2 is the most abundant TGF-β isoform in human atherosclerotic carotid plaques. *A*) Plots illustrating the vast differences in human carotid plaque levels of TGF-β1, -β2, and -β3. Lines and bars indicate median and inter quartile range (IQR). ****P < 0.0001. Kruskal–Wallis and Mann–Whitney *U* tests were used to determine levels of significance. Values on the *y*-axis indicate levels of the respective component in pg/g wet weight plaque. *n* = 223. *B*) Immunohistochemistry of TGF-β1, TGF-β2, and TGF-β3 on adjacent slides from human atherosclerotic plaque (*n* = 5). Scale bars represent 300 µm. CTL, control.

Analysis of human carotid plaques revealed that all three isoforms of TGF-β were detected and that TGF-β2 was more abundant than TGF-β1 and -β3 (see Supplementary material online, *Table S3* and *Figure 1A*; *P* < 0.0001). Interestingly, median plaque levels of TGF-β2 [1261.15 (802.40–2252.48) pg/g] were 7 times higher than those of TGF-β1 [181.96 (97.37–346.95) pg/g] and 20 times higher than those of TGF-β3 [61.64 (34.27–108.84) pg/g]. The presence of TGF-β1, TGF-β2, and TGF-β3 in human plaque tissue was also confirmed by immunohistochemistry (*Figure 1B*).

Next, TGF-β recovery from spiked plaque homogenates was assessed to confirm the accuracy of our protein measurements. According to our recovery trials, ∼60–70% of the three isoforms were recovered and no significant differences between the recovery of the three isoforms were detected (see Supplementary material online, *Figure S1*).

### 3.2 TGF-β2 is a main component of asymptomatic carotid plaques

**Figure 2:**
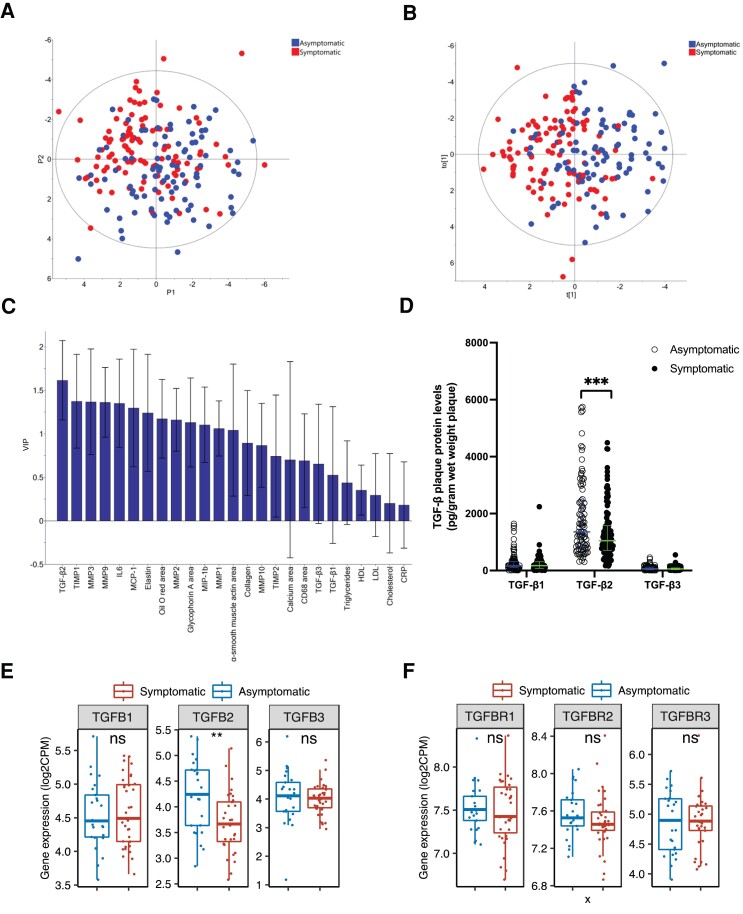
TGF-β2 is increased in asymptomatic plaques and is the main determinant separating asymptomatic and symptomatic plaques. *A*) Asymptomatic plaques were separated from symptomatic plaques by an unsupervised principal component analysis. Asymptomatic plaques marked in blue and symptomatic plaques marked in red. *B*) The main determinant biological components driving the separation of asymptomatic plaques from symptomatic plaques were identified by an OPLS-DA analysis and according to *C*) the VIP-score analysis the most important component was TGF-β2. *D*) Plot representing the three different isoforms of TGF-β (-β1, -β2, and -β3) measured in plaque homogenate comparing plaques from asymptomatic vs. symptomatic patients. Only TGF-β2 was significantly higher in plaques from asymptomatic patients compare to plaques from symptomatic patients (P = 0.003). Group comparisons were performed with Mann–Whitney’s test. Lines and bars represent median and inter quartile range (IQR). *E*). Box plots showing mRNA levels of the three isoforms of TGFB (-B1, -B2, and -B3) and *F*) their receptors TGBR1, TGFBR2, and TGFBR3 measured in a subset of 60 plaques. Boxes indicate interquartile range. Center line indicates median. Whiskers indicate minimum (within lower quartile −1.5 times interquartile range) to maximum (within upper quartile + 1.5 times interquartile range). Blue bars indicate asymptomatic and red bars indicate symptomatic plaque levels. **, P ≤ 0.01 with Students t-test.

As TGF-β has been suggested to be important for tissue repair, we aimed to identify if any of the three TGF-β isoforms was associated with asymptomatic plaque phenotype. To test this, we performed a PCA of the assessed TGF-β isoforms abundances, inflammatory cytokines, MMPs, TIMPs, histological markers of plaque stability and vulnerability, ECM key proteins, and circulating lipoproteins. Interestingly, PCA analysis showed a separation between symptomatic (from patients who suffered from a stroke, AF, or TIA within a month before surgery) and asymptomatic plaques (*Figure 2A*). Subsequently, a supervised OPLS-DA was performed to improve sample separation observed in the PCA model, as the first. Subsequently, OPLS-DA was performed as it has greater potential of identifying the most relevant variables for separation of groups (*Figure 2B*; the variable contribution is presented in Supplementary material online, *Table S4*). Importantly, TGF-β2 was the strongest biological component for segregating asymptomatic plaques from symptomatic plaques (*Figure 2C*), suggesting that TGF-β2 contributes to a stable plaque phenotype.

Moreover, when comparing TGF-β levels in symptomatic plaques with asymptomatic plaques, TGF-β2 levels were significantly higher in asymptomatic plaques [1435.77 (845.59–2758.60) pg/g vs. 1047.71 (710.34–1573.26) pg/g, *P* = 0.003; *Figure 2D*]. TGF-β1 and TGF-β3 protein levels did not differ between symptomatic and asymptomatic plaques. The mRNA levels of TGFB1, -B2, and -B3 were also assessed in a subgroup of 60 plaques using RNA sequencing. In line with the protein levels, TGFB2 mRNA levels were higher in asymptomatic plaques compared to symptomatic plaques (*Figure 2E*, *P* = 0.009). No differences were found for the other two isoforms, TGFB1 or TGFB3, corroborating the findings found at the protein level. To confirm differences in mRNA expression levels, carotid plaque gene expression of the three TGFB isoforms was measured in the validation cohort (BiKE cohort) by microarrays. Importantly, again TGFB2 mRNA expression was found to be significantly higher in asymptomatic plaques compared to symptomatic plaques, Supplementary material online, *Figure S2D*.

Next, asymptomatic plaques were shown to have a more stable plaque phenotype than symptomatic plaques with smaller plaque areas of CD68 [22.8 (IQR14.2–31.9) vs. 26.9 (IQR 18.2–35.1) percent of total plaque area; *P* = 0.01], Oil Red O [23.1 (IQR14.2–32.9) vs. 30.0 (IQR 18.7–39.5) percent of total plaque area; *P* = 0.003] and glycophorin A [3.9 (IQR1.9–9.1) vs. 7.2 (IQR 3.6–12.7) percent of total plaque area; *P* = 0.002] as well as larger plaque areas of collagen [2.6 (IQR1.1–8.1) vs. 1.9 (IQR 0.7–4.5) percent of total plaque area; *P* = 0.03] and smooth muscle alpha-actin [26.0 (IQR17.7–36.0) vs. 18.0 (IQR 12.3–30.2) percent of total plaque area; *P* = 0.0018] compared to symptomatic plaques (see Supplementary material online, *Figure S3A*). Also, a calculated histological plaque VI, previously used to identify unstable plaques and to predict future CV events,^16,27,28^ was shown to be significantly lower in asymptomatic plaques [VI 1.6 (IQR 1.0–2.8) vs. 2.8 (IQR 1.6–4.9), *P* < 0.001; Supplementary material online, *Figure S3B*], all in support for a more stable plaque phenotype in asymptomatic plaques.

As the biological effect of the three TGF-β isoforms is not solely dependent on protein levels but also on their functional receptors, gene expression levels of the three major TGF-β receptors (TGFBR1/-R2/-R3) in the human plaques were assessed by mRNA sequencing. However, no significant differences were detected when comparing patients with or without symptomatic disease (*Figure 2F*). Neither did plaque protein or mRNA levels of endoglin (part of the TGF beta receptor complex) show any differences between symptomatic and asymptomatic plaques (see Supplementary material online, *Figure S4A and B*). As both free protein levels and mRNA levels of TGF-β2 were reduced in symptomatic plaques, these findings together point towards the importance of reduced TGF-β2 levels rather than a TGF-β2 signalling inability in itself.

### 3.3 TGF-β2 is dependent on the presence of vascular smooth muscle cells

To investigate why the TGF-β2 isoform was exclusively elevated in asymptomatic plaques, the CARDIoGRAMplusC4D consortium and GTEx databases were used to screen for potential single-nucleotide polymorphisms (SNPs) associated with CV disease and TGFβ2 expression. However, no genome-wide significant SNPs were identified, suggesting that this finding is less likely due to genetic differences but rather cellular changes associated with TGF-β2 expression. Protein levels were correlated to the plaque area of the smooth muscle cell marker alpha-smooth muscle actin and the macrophage marker CD68. Interestingly, only TGF-β2 correlated to alpha-smooth muscle actin^+^ plaque area (*Table 1*). In line with this, TGF-β2^+^ areas were identified in close proximity to alpha-smooth muscle actin^+^ areas in the fibrous cap (see Supplementary material online, *Figure S5A*) and immunofluorescence visualised the expression of TGF-β2 on alpha-smooth muscle actin^+^ cells (see Supplementary material online, *Figure S5B*). In further support, alpha-smooth muscle actin^+^ plaque area was significantly smaller in symptomatic plaques (symptoms less than 1 month prior to surgery) compared to asymptomatic plaques (see Supplementary material online, *Figure S3A*).

**Table 1:** Spearman correlations between the three isoforms of TGF-β (-β1, -β2, and -β3) and the histologically assessed plaque components

Histological component	TGF-β1		TGF-β2		TGF-β3	
	*r*	*q*	*r*	*q*	*r*	*q*
α-smooth muscle actin	0.088	0.399	0.262	0.000042	0.141	0.076
Calcium	−0.187	0.025	−0.154	0.006	−0.190	0.021
Oil Red O	−0.037	0.616	−0.178	0.003	0.008	0.815
CD68	−0.068	0.438	−0.133	0.01	−0.003	0.815
Glycophorin A	0.003	0.808	−0.329	0.000003	−0.088	0.28

Correlation analyses between plaque levels of TGF-β isoforms and histologically assessed plaque components identified positive correlations between TGF-β2 and plaque area of α-smooth muscle actin (smooth muscle cells) as well as inverse correlations to plaque area of CD68 (macrophages), glycophorin A (intraplaque haemorrhage), and Oil Red O (neutral lipids). The data were analyzed using the Spearman correlation coefficient. All statistical *P*-values were corrected for multiple comparisons using the two-stage step-up method of Benjamin, Krieger, and Yekutieli with a false discovery rate <5% and are reported as *q*-values. *n* = 196–223.

### 3.4 TGF-β2 is associated with features of plaque stability

To further study if TGF-β2 was associated with biological components contributing to plaque stability, a *K*-means clustering (*K* = 2) was performed based on the 11 factors contributing to the separation of asymptomatic plaques in the OPLS analysis (VIP-score >1, *Figure 2C*).

**Figure 3:**
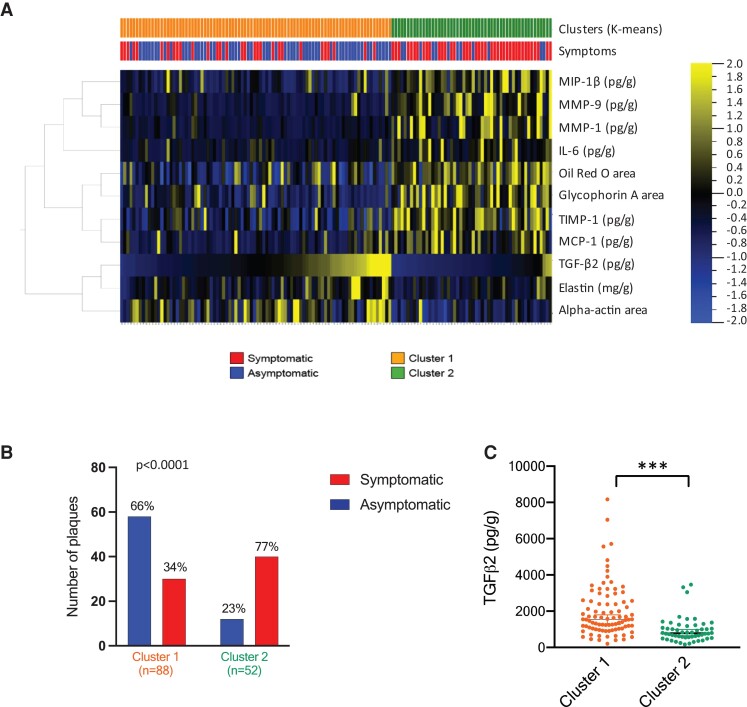
Higher plaque levels of TGF-β2 are associated with elastin, smooth muscle cells and asymptomatic plaques. *A*) An unsupervised K-means clustering based on the 11 main components in the VIP-score analysis identified two main clusters, cluster 1 and cluster 2. Plaques in cluster 1 had *B*) a significant higher percentage of asymptomatic plaques compared to cluster 2. *n* = 88 for cluster 1 and *n* = 52 for cluster 2. Cluster 1 also had more smooth muscle cell alpha-smooth muscle actin (% of plaque area), elastin (mg/g plaque wet weight) and *C*) more TGF-β2 (pg/g wet weight plaque) compared to cluster 2. ***, *P* ≤ 0.001.

Using this approach, we identified two clusters (*Figure 3A*). Plaques in cluster 1 had high levels of TGF-β2, elastin, and larger plaque areas stained positive for alpha-smooth muscle actin compared to plaques in cluster 2. Cluster 2 plaques had instead higher levels of MMPs, inflammatory cytokines, lipids, and more intraplaque hemorrhage (*Figure 3A*). Furthermore, cluster 1 had significantly more asymptomatic plaques compared to cluster 2 (*Figure 3B*) and significantly higher levels of TGF-β2 compared to cluster 2 (*Figure 3C*).

When exploring how TGF-β2 levels correlated with specific biological components, a positive correlation was found with alpha-smooth muscle actin content (*r* = 0.262, adjusted *q*-value = 4 × 10^−5^). Inverse correlations were found with macrophage CD68^+^ plaque area (*r* = -0.133, adjusted *q*-value = 0.01), neutral lipids (Oil red O; *r* = -0.178, adjusted *q*-value = 0.003), and glycophorin A (*r* = -0.329, adjusted *q*-value = 3 × 10^−6^; *Table 1*), a marker of intraplaque haemorrhage. TGF-β1 and TGF-β3 levels only correlated inversely to plaque calcium area (*r* = -0.187, adjusted *q*-value = 0.025 and *r* = -0.190, adjusted *q*-value = 0.021; *Table 1*). Even though the correlations coefficients are not very strong, this suggests that TGF-β2 is important for plaque stability since smooth muscle cells are the main producers of ECM proteins in plaques. Also, the inverse correlations to lipids, CD68^+^ and intraplaque haemorrhage^+^ areas, which are all associated with the risk for a plaque rupture, further corroborates the association of TGF-β2 and a stable plaque phenotype.

To evaluate the role of the TGF-β isoforms in plaque remodeling, several MMPs (-1, -2, -3, -9, and -10) and TIMPs (1 and 2) were measured in plaque homogenates using a multiplex assay. Even if not very strong, but still significantly, TGF-β2 correlated inversely with MMP-9 and TIMP-1 and positively to MMP-2, MMP-3, and TIMP-2 (*Table 2*). TGF-β1 did not correlate with any MMPs or TIMPs, whereas TGF-β3 displayed an association to MMP-2 (*Table 2*).

**Table 2:** Spearman correlations between the three isoforms of TGF- β (-β1, -β2, and -β3) and matrix metalloproteinase (MMP)-1, -2, -3, -9 -10, and the tissue inhibitors of metalloproteinases (TIMP)-1 and -2

MMPs/TIMPS	TGF-β1		TGF-β2		TGF-β3	
	*r*	*q*	*r*	*q*	*r*	*q*
MMP-1	−0.008	0.95	−0.134	0.02	0.064	0.56
MMP-2	0.163	0.13	0.208	0.002	0.311	0.00003
MMP-3	0.115	0.36	0.168	0.006	0.130	0.15
MMP-9	0.031	0.95	−0.310	0.000007	0.019	0.88
MMP-10	−0.008	0.95	−0.021	0.23	0.002	0.88
TIMP-1	−0.016	0.95	−0.227	0.0007	−0.002	0.88
TIMP-2	−0.047	0.95	0.309	0.000007	0.127	0.15

Correlation analyses between plaque levels of TGF-β isoforms and plaque levels of MMPs and TIMPS identified positive correlations between TGF-β2, MMP-2/-3, and TIMP-2 as well as inverse correlations to MMP-1/-9 and TIMP-1. The data were analyzed using the Spearman correlation coefficient. All statistical *P*-values were corrected for multiple comparisons using the two-stage step-up method of Benjamin, Krieger, and Yekutieli with a false discovery rate <5% and are reported as *q*-values. MMP, Matrix metalloproteinases; TIMPS, Tissue inhibitors of matrix metalloproteinases. *n* = 202–208.

**Table 3:** Spearman correlations between the three isoforms of TGF-β (-β1, -β2, and -β3) and extracellular matrix components glycosaminoglycans, collagen and elastin

ECM Components	TGF-β1		TGF-β2		TGF-β3	
	*r*	*q*	*r*	*q*	*r*	*q*
Glycosaminoglycans	0.269	0.017	0.331	0.002	0.301	0.004
Collagen	0.240	0.002	0.340	8.1 × 10^−7^	0.279	7 × 10^−5^
Elastin	0.140	0.047	0.394	1.4 × 10^−8^	0.300	3.8 × 10^−5^

All three TGF-β isoforms were positively correlated to plaque levels of glycosaminoglycans, collagen and elastin. The data were analyzed using the Spearman correlation coefficient. All statistical *P*-values were corrected for multiple comparisons using the two-stage step-up method of Benjamin, Krieger, and Yekutieli with a false discovery rate <5% and are reported as *q*-values. *n* = 88 (glycosaminoglycans) and *n* = 206–208 for elastin and collagen.

The TGF-β isoforms are well known to induce the synthesis of several ECM proteins including collagens and elastin,^29–31^ which are essential for plaque stabilization. To investigate the relation between the three TGF-β isoforms and the ECM components in the plaque, we measured biochemically the main ECM components in the plaque tissue, namely GAG, collagen, and elastin. All three isoforms of TGF-β were positively correlated to the three ECM components (*Table *3**). Furthermore, the levels of TGF-β isoforms were correlated to the time between symptoms and surgery (see Supplementary material online, *Table S5*). This suggests that TGF-β isoforms increase over time and possibly reflect an ongoing beneficial repair process in the plaque after a symptom-causing plaque rupture.

### 3.5 Lower plaque levels of TGF-β2 are associated with lower levels of target proteins

To elucidate if reduced plaque levels of TGF-β2 were also reflected by lower biological activity, two target proteins downstream of TGF-β2 signaling, PAR-1 and MMP-3, were measured in plaque tissue homogenates.^32,33^ TGF-β2 correlated to both PAR-1 and MMP-3 plaque protein levels (*r* = 0.66, *P* < 0.001 and *r* = 0.17, *P* = 0.017, respectively; Supplementary material online, *Table S6*). In line with TGF-β2 plaque levels, both PAR-1 and MMP-3 plaque protein levels were significantly higher in asymptomatic plaques compared to symptomatic plaques (see Supplementary material online, *Figure S6*).

### 3.6 TGF-β2 is associated with reduced plaque inflammation and inflammatory activity *in vitro*

**Figure 4:**
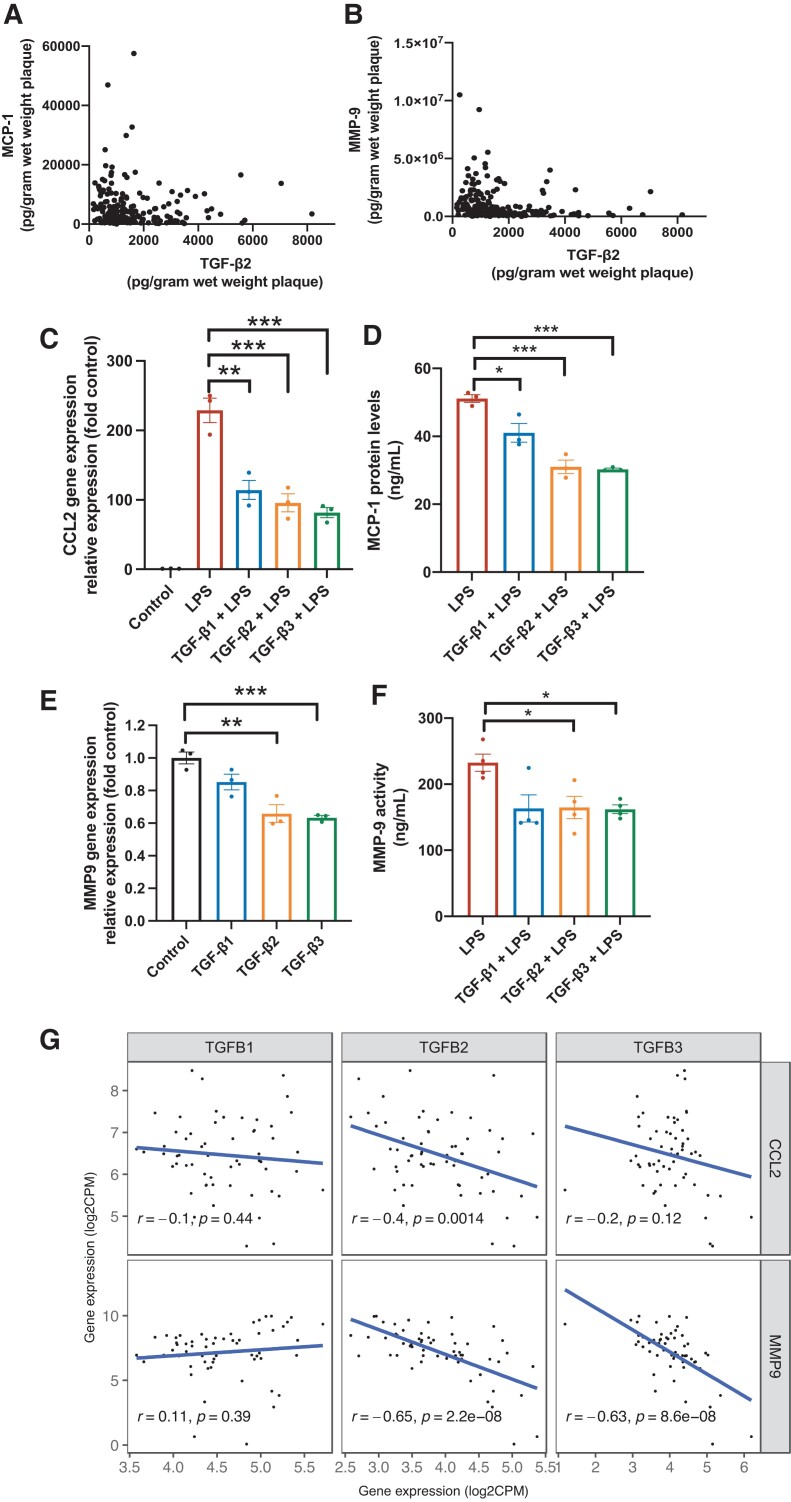
TGF-β2 is associated with lower levels of inflammatory markers and decreases the inflammatory response in THP-1 macrophages. *A*, *B*) Scatterplots showing correlations of TGF-β2 plaque levels and plaque levels of monocyte chemoattractant protein-1 (MCP-1, *n* = 181) and matrix metalloproteinase-9 (MMP-9, *n* = 208). Spearman’s correlation test was used. *C*) TGF-β pre-stimulation (5 ng/mL) *in vitro* decreased CCL2 (MCP-1) gene expression (quantified by qPCR, *n* = 3) in LPS treated (100 ng/mL), PMA activated THP-1 cells. ANOVA and Student’s *t*-tests were used. *D*) All three TGF-β isoforms reduced free MCP-1 protein levels in supernatants collected from LPS stimulated PMA matured THP-1 macrophages (*n* = 3). Student’s *t*-test was used. *E*) TGF-β2 and TGF-β3 pre-stimulation (5 ng/mL) decreased MMP9 gene expression (quantified by qPCR, *n* = 3) in PMA activated THP-1 cells. ANOVA and Student’s *t*-tests were used. *F*) TGF-β2 and TGF-β3 reduced MMP-9 activity in PMA activated (100 ng/mL) THP1 cells upon LPS stimuli (1 µg/mL) for 24 h. *n* = 3. Kruskal–Wallis and Mann–Whitney *U* tests were used. *G*) Plaque TGFB2 mRNA levels correlated inversely to CCL2 (MCP-1) and MMP9 mRNA levels in human carotid plaques. Pearson’s correlation test was used (*n* = 60). *, *P* < 0.05, ** *P* < 0.01, ***P < 0.001.

Since TGF-β1 is known to reduce the inflammatory response, we assessed if TGF-β2 also could affect the inflammatory process that contributes to a rupture-prone plaque phenotype. In plaque homogenates, TGF-β2 correlated inversely to the pro-inflammatory cytokine monocyte chemoattractant protein 1 (MCP-1) (*r* = -0.178 *P* = 0.017) and MMP-9 (*r* = -0.310, *P* = 0.0001, *Figure 4A* and *B* and *Table 2*). To assess the anti-inflammatory properties of TGF-β2, PMA-activated THP-1 cells (assigned to as M0 macrophages) were stimulated with 5 ng/mL TGF-β1, -β2, and -β3 for 48 h. Subsequently, cells were stimulated with 100 ng/mL LPS overnight (to induce an M1-like pro-inflammatory macrophage phenotype). In LPS-stimulated cells, CCL2 (MCP-1) gene expression was reduced upon treatment with TGF-β1, TGF-β2, and TGF-β3 by 50% (*P* = 0.007), 58% (*P* = 0.004), and 64% (*P* = 0.001), respectively (*Figure 4C*), as well as MCP-1 protein secretion (TGF-β1 20% (*P* = 0.03); TGF-β2 39% (*P* = 0.001); TGF-β3 41% (*P* < 0.001), respectively), *Figure 4D*.

Furthermore, TGF-β treatment reduced MMP9 gene expression. TGF-β2 caused a 34% reduction (*P* = 0.007), and TGF-β3 caused a 37% reduction in MMP9 gene expression compared to control (*P* = 0.0007, *Figure 4E*). TGF-β2 and TGF-β3 also reduced MMP-9 activity, measured in the cell culture supernatants upon LPS stimuli *Figure 4F*). No significant effect on MMP-9 gene expression and activity by TGF-β1 treatment was identified.

Next, we explored if TGF-β treatment would affect the inflammatory response induced by oxidized LDL. Again, all three isoforms of TGF-β significantly reduced CCL2 mRNA levels (see Supplementary material online, *Figure S7A*), whereas only TGF-β1 and -β3 had a significant effect on MMP9 mRNA levels (see Supplementary material online, *Figure S7B*). However, oxidised LDL only induced a minor MMP9 response *in vitro*, suggesting that other factors have possibly a larger impact on MMP-9 expression. Additionally, plaque levels of oxidized LDL were only associated to plaque levels of MCP-1 (*r* = 0.36, *P* < 0.001) and not MMP-9 (*r* = 0.1, *P* = 0.1).

In line with the *in vitro* studies, additional RNAseq analyses on human plaque tissue identified inverse correlations between mRNA levels of TGFB2/B3, CCL2, and MMP9, whereas no such correlations were identified between TGFB1 and CCL2 or MMP9 *Figure 4G*). These inverse correlations of mRNA levels were confirmed in a similar, independent, endarterectomy cohort, the BiKE cohort (see Supplementary material online, *Figure S8*).

Finally, additional experiments using the murine microphage cell line, RAW264.7 cells, confirmed the inflammation dampening effect of TGF-β treatment on LPS-activated macrophages: CCL2 expression was reduced by TGF-β2 by 13% (*P* = 0.02) and by TGF-β3 by 34% (*P* < 0.001). MMP9 expression was reduced by 26% (*P* < 0.001) and 84% (*P* < 0.001) after treatment with TGF-β2 and TGF-β3 (see Supplementary material online, *Figure S9*). In opposite, TGF-β1 treatment was shown increase both CCL2 and MMP9 gene expression (*P* < 0.005).

### 3.7 High levels of TGF-β2 in the plaque are associated with reduced risk of future CV events

**Figure 5:**
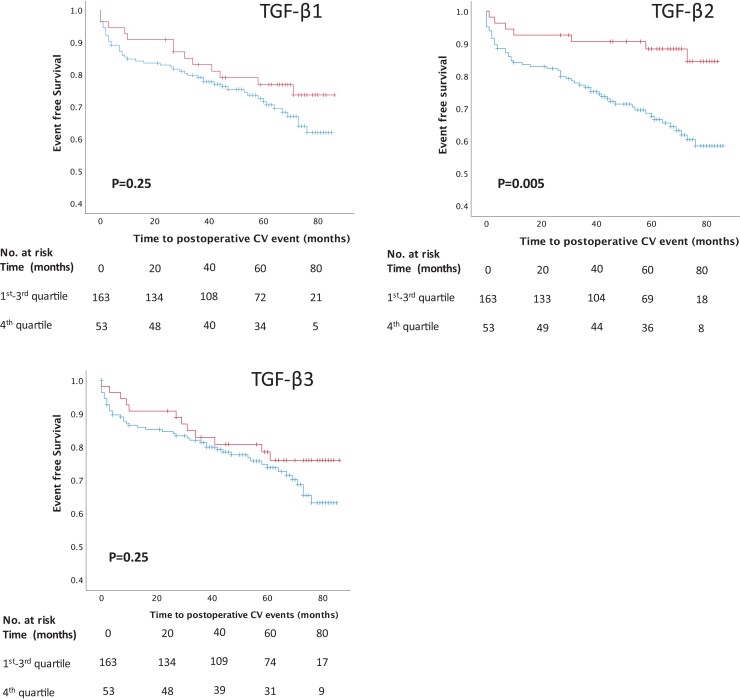
Patients with high plaque levels of TGF-β2 have a lower risk for future cardiovascular events. Kaplan–Meier curves showing cumulative incidence of cardiovascular events. Patients with TGF-β2 levels in the highest quartile (red) have significantly lower risk to suffer from future cardiovascular events compared to patients in 1st-3rd quartile (blue). No significant differences were identified for TGF-β1 and TGF-β3.

Since TGF-β2 levels were associated with a stable plaque phenotype and elevated in asymptomatic plaques, it was of clinical interest to study if TGF-β2 levels were related to future CV events. Despite being a relatively small study for risk prediction, we found that high TGF-β2 protein levels {highest quartile; median TGF-β2 protein levels [median TGF-β2 protein levels 3216 pg/gram wet weight plaque (IQR 2685–4336 pg/g)]} were associated with a lower risk of future CV events during the follow-up period (Log-rank test with *P* = 0.005, *Figure 5*) compared to the combined 1st-3rd quartile [median TGF-β2 protein levels 1008 (IQR 676–1419 pg/gram wet weight plaque)], whereas no significant differences were detected for TGF-β1 or TGF-β3 (*Figure 5*). In the group with TGF-β2 levels in the highest quartile, 7 out of 54 (13%) patients suffered from CV events, whereas in the group of patients with TGF-β2 levels in the three lowest quartiles, 51 out of 164 suffered from CV event (31%).

A multivariable Cox regression model adjusted for age, sex, hypertension, total cholesterol, HDL, and current smoking showed that the highest quartile of TGF-β2 remained associated with a lower risk for future CV events with a HR of 0.41 (95% CI 0.2–0.8, *P* = 0.02; Supplementary material online, *Table S7*) compared to the lowest quartile. Furthermore, 1 SD increase in TGF-β2 was associated with a lower risk of future CV events [HR 0.69 (95% CI0.52–0.93, *P* = 0.02)].

Finally, to explore the effect of multiple comparisons for the survival analysis we randomly shuffled the exposures (TGF-β2 levels) 1000 times and performed Cox-regressions consequently. This would report 1000 statistic values (i.e. *Z* scores) and their distribution. We then examined the *Z* score from the unshuffled TGF-β2 in that distribution. This permutation test gave a *P*-value of 0.005 comparing the 4th quartile vs. 1st–3rd quartiles. A *P*-value of 0.002 was obtained if treating TGF-β2 as continuous values (see Supplementary material online, *Figure S10*). Both results suggested that the divergent curves were less likely to be random events.

## 4. Discussion

The present study is the first to quantify all three TGF-β isoforms (-β1, -β2, and -β3) in human atherosclerotic tissue^34,35^ and to show that TGF-β2 is the most abundant isoform. TGF-β2 was identified as a main component in asymptomatic plaques and correlated to smooth muscle cell content, as well as the expression of stabilizing ECM proteins. TGF-β2 was also inversely associated with important factors for plaque rupture, namely inflammation, intraplaque haemorrhage and MMP-9, and it decreased important chemokines and protease activity *in vitro*. Additionally, low plaque levels of TGF-β2 predicted CV events together with age. Together indicating protective role of TGF-β2 in atherosclerotic complications which has not previously been studied.

CV events and death are commonly caused by MI or stroke due to atherosclerotic plaque ruptures. Rupture-prone plaques are known to have less stabilizing ECM proteins and thinner protective caps with more collagen degradation due to increased MMP-9 and higher inflammatory activity.^36^

The present study provides evidence for an important role of TGF-β2 in plaque stability. First, we identified TGF-β2 as the main component for the separation of asymptomatic plaques from symptomatic plaques in our analysis. Next, the reduced levels of TGF-β2 found in plaques associated with symptoms are in line with the notion that TGF-β2 contributes to plaque stability. TGF-β2 is important for ECM remodeling in the embryonic development of CV structures,^37,38^ but its role in ECM formation and remodelling in atherosclerosis has sparsely been investigated. According to the present study, TGF-β2 is associated with increased levels of collagen and vascular smooth muscle cells in plaques. Collagens are mainly produced by vascular smooth muscle cells and are suggested to stabilize the plaque and prevent ruptures.^39^

Increased levels of collagen could imply a reduced degradation of collagen. MMP-9 is important in collagen degradation and subsequent risk for plaque rupture.^40^*In vitro* experiments have shown that TGF-β1 can suppress levels of MMP-9,^41^ although it is not known if this applies to TGF-β2. In the present study, we show that TGF-β2 protein and mRNA levels are inversely associated with MMP-9 protein and mRNA levels in the human plaque tissue. This suggests that also TGF-β2 acts as a suppressor of MMP-9, which would in turn reduce collagen degradation. This is further corroborated by our *in vitro* findings showing that TGF-β2 decreases MMP9 mRNA levels in THP-1 derived macrophages and even more importantly, TGF-β2 was found to reduce MMP-9 activity in THP-1 derived macrophages.

The effect of TGF-β2 on MMP activity is of interest considering its selective upregulation in atherosclerotic plaques, with 7–20-fold higher protein levels compared to TGF-β1 and TGF-β3. MMPs are known to be master regulators of TGF-β complex cleavage, especially MMP-2 and MMP-9. However, the capacity to activate each TGF-β isoform may vary between MMPs as well as plaque levels of each MMP.^42^ In support of a potential effect of plaque MMP levels, we have previously shown that MMP-9 is the most abundant MMP isoform in human carotid plaques.^20^ Therefore, a potential mechanism underlying a selective increase in TGF-β2 plaque levels could be high plaque levels of MMP-9, which could favour a greater TGF-β2 release and, in turn, downregulate MMP-9 as a negative feedback mechanism. Importantly, no major differences in the recovery of the three TGF-β isoforms were detected when spiking human plaque samples with recombinant proteins, showing that the selective upregulation of TGF-β2 was not caused by technical issues.

A prominent feature of rupture-prone plaques is inflammation. TGF-β1 has been shown to have strong anti-inflammatory properties whereas less is known about TGF-β2. In plaque tissue, we found an inverse association to the well-known pro-inflammatory cytokine MCP-1. Moreover, our *in vitro* studies showed reduced CCL2 gene expression, as well as a reduced secretion of MCP-1 from THP-1 cells pre-stimulated with TGF-β2 before LPS activation, which further emphasizes that TGF-β2 dampens the inflammatory response. Even though a similar suppressive effect was identified for TGF-β3, the biological effect of TGF-β2 is likely greater considering the 20-fold higher content of TGF-β2. This is an important finding as MCP-1 acts as a strong chemoattractant for monocytes/macrophages.^43^ In line with the suppressive effect of TGF-β2 on MCP-1 *in vitro*, TGF-β2 plaques levels were negatively correlated to plaque CD68 expression, suggesting a reduced migration of myeloid cells into the plaque due to reduced MCP-1 levels.

Finally, we aimed to assess if TGF-β isoforms can predict future CV events. Importantly, patients in the group with TGF-β2 levels in the highest quartile had significantly lower risk for CV events, which was also confirmed in a multivariate Cox regression analysis. The reduced levels of TGF-β2 found in the symptomatic group may not only be a response to a recent plaque rupture, but also an indicator of an impaired repair response, associated with a worse outcome. Interestingly TGF-β1 and -β3 levels were not associated with a lower risk of CV events.

TGF-β2 protein levels measured in plaque tissue are difficult to use as a clinical biomarker, therefore these associations rather emphasize the potential biological role on plaque stability. Since the recorded CV events may originate from other atherosclerotic plaques than where TGF-β2 levels were measured (in this case the surgically removed carotid plaque), this indicates that the impaired repair response might not only be a local finding but rather reflect a systemically impaired repair response.

In further support for the role of TGF-β in repair responses, all three isoforms were positively correlated to the time since the event of a plaque rupture (*r* = 0.26, *P* = 0.004; *r* = 0.21, *P* = 0.02 and *r* = 0.29, *P* = 0.001, respectively), fitting nicely with the *in vitro* studies. This indicates that TGF-β2 becomes progressively more abundant after rupture, likely reflecting a beneficial healing response, which is also associated with cellular phenotype changes.

Monocyte-derived macrophages are a dominant immune cell population in atherosclerotic plaques and monocyte-infiltration is likely boosted upon a CV event.^44^ As part of the repair process initiated to counterbalance the inflammatory process upon a plaque rupture, macrophages are thought to be alternatively activated, undergo a phenotype shift and become more ‘M2’-like (anti-inflammatory). This macrophage phenotype will contribute to the resolution of inflammation and thus express TGF-β1.^45^ Herein, it is likely that the relatively fast phenotype shift upon a rupture will induce a rapid TGF-β1 response, whereas the migration of TGF-β2-associated smooth muscle cells will be a slower process. In support of this, our immunohistochemically analyses identified TGF-β2^+^ areas in close proximity to the smooth muscle cells in the fibrous cap, whereas TGF-β1 was staining showed a more general pattern, co-localizing with both CD68^+^ and alpha-smooth muscle actin^+^ areas. These findings suggest that smooth muscle cells are the main cellular source of TGF-β2 in human atherosclerosis. This notion is corroborated by a greater alpha-smooth muscle actin^+^ plaque area in asymptomatic plaques.

### 4.1 Limitations

It should be considered that the study is based on samples from a cohort with advanced atherosclerotic disease and the results may not reflect the early stages of atherosclerosis. Furthermore, even though the results are corroborated *in vitro*, *in situ*, and by follow-up data, the present study does not provide a definite proof of causality. Some of the correlations obtained do not have a very strong correlation coefficient, which may demand some caution in the interpretation. However, this is common in human tissues with large biological variation. Additionally, the *P-*values are provided and adjusted for multiple comparisons when needed. Protein measurements of TGF-β isoforms examined in this study may potentially also reflect the latent form, however the plaque homogenates were not acidified during the analysis to avoid the release of latent TGF-β. Further studies are needed to find ways of manipulating this novel pathway in a specific manner to increase plaque stability.

It should also be considered that all patients included in the current study suffer from advanced carotid stenosis. It should be noted that asymptomatic patients accepted for surgery commonly suffer from a greater degree of stenosis (>80%) compared to the symptomatic (>70%) to compensate for the surgical risk in a primary prevention intervention. Therefore, the risk factor pattern is different, e.g. being smoking and hypertension more common among patients without symptomatic disease.

The use of alpha-smooth muscle actin as a marker of smooth muscle cells in atherosclerosis is complicated by the fact that ∼40% of these cells are also positive for CD68 positive in human coronary atherosclerotic plaques.^46,47^ Yet, smooth muscle cells present in the cap, producing collagen and contributing to plaque stability have, in contrast to the smooth muscle cells closer to the media, been shown to strongly express alpha-actin. Also, smooth muscle cells expressing CD68 are suggested to become more ‘macrophage-like’, able to induce inflammatory responses and to engulf lipids. In the present study, no associations between alpha-smooth muscle actin plaque area and plaque levels of pro-inflammatory cytokines IL-6 or MCP-1 (*r* = -0.14, *P* = 0.06 and *r* = -0.12, *P* = 0.1) were identified. In contrast, CD68 plaque area correlated positively to MCP-1 protein level (*r* = 0.24, *P* = 0.001). No positive correlation between plaque area stained positive for alpha-smooth muscle actin and CD68 (*r* = -0.13, *P* < 0.05) was identified either. Additionally and in agreement with these results, the co-localization of alpha-smooth muscle actin and CD68 on plaque tissue sections was sparse.

In summary, the findings in the present study indicate that TGF-β2 has a potential role in atherosclerotic repair mechanisms and contributes to a less rupture-prone plaque phenotype. Even though further studies are needed, these findings together indicate a novel and previously unexplored protective role of TGF-β2 in atherosclerotic disease.

## Supplementary material

Supplementary material is available at *Cardiovascular Research* online.

## Supplementary Material

cvad079_Supplementary_Data

## Data Availability

The dataset is not publicly available due to the sensitive nature of the data regulated by GDPR regarding living subjects. Sensible requests to access the dataset from qualified researchers trained in human subject confidentiality protocols may be sent to Prof Isabel Goncalves at Lund University.

## Notes

Translational perspective The balance between inflammation and the resolution of inflammation is considered to be key for atherosclerotic plaque ruptures and subsequent clinical complications such as heart attacks and strokes. However, which biological factors halt human atherosclerotic plaque inflammation remains to be explored. In the present study, TGF-β2 is associated with stable plaques, less cardiovascular (CV) complications and reduces the release of the pro-inflammatory cytokine MCP-1 and matrix degrading protease MMP-9. Together, these findings suggest that TGF-β2 could be used to stabilize rupture prone plaques by reducing inflammation and extracellular matrix degradation.
