# Supplementary material for: Transforming growth factor-β2 is associated with atherosclerotic plaque stability and lower risk for cardiovascular events
Source: Cardiovasc Res. 2023 May 18;119(11):2061–73. doi: 10.1093/cvr/cvad079 (PMC10478752; doi:10.1093/cvr/cvad079)
Supplement: cvad079_Supplementary_Data [file cvad079_supplementary_data.zip › Low_TGF_Suppl_202230123.docx]

**SUPPLEMENTAL MATERIAL**

**Increased transforming growth factor-****β2 is associated with human atherosclerotic plaque stability and lower risk for cardiovascular events**

**Authors:** Andreas Edsfeldt^1,2,3,^ Pratibha Singh^1^, Frank Matthes^1^, Christoffer Tengryd^1^, Michele Cavalera^1^, Eva Bengtsson^1,4,5^, Pontus Dunér^1^, Petr Volkov^6,7^, Glykeria Karadimou^8^, Anton Gisterå^9^, Marju Orho-Melander^1,10^, Jan Nilsson^1^, Jiangming Sun ^1^ and Isabel Gonçalves^1,3^

**Affiliations:**

^1^Department of Clinical Sciences, Malmö, Lund University, Sweden.

^2^Wallenberg Center for Molecular Medicine, Lund University, Sweden

^3^Department of Cardiology, Skåne University Hospital, Sweden

^4^ Faculty of Health and Society, Malmö University, Malmö, Sweden

^5^Biofilms – Research Center for Biointerfaces, Malmö University, Malmö Sweden

^6^LUDC Bioinformatics Unit, Department of Clinical Sciences, Malmö, Lund University, Sweden

^7^Data Science and Quantitative Biology, Discovery Sciences, R&D, AstraZeneca, Gothenburg, Sweden.

^8^Department of Molecular Medicine and Surgery, Karolinska Institute, Stockholm, Sweden

^9^Center for Molecular Medicine, Department of Medicine, Solna, Karolinska University Hospital, Karolinska Institutet, Stockholm, Sweden

^10^Diabetes and Cardiovascular Disease Genetic Epidemiology Unit, Department of Clinical Sciences, Malmö, Lund University, Sweden.

**Correspondence**

^*^Andreas Edsfeldt, Clinical Research Center, Jan Waldenströms gata 35, 91:12, Skåne University Hospital, SE-20502 Malmö, Sweden. Tel +46-40-391238 Fax +46-40-391212. E-mail: Andreas.edsfeldt@med.lu.se

**Supplementary methods**

*Study design*

223 human carotid plaques were obtained from 218 patients who underwent carotid endarterectomy between 2005 and 2011 at the Vascular Department at Skåne University Hospital (Malmö, Sweden). Five patients underwent surgery for both right and left carotid plaques. All the patients gave written and oral informed consent. The study was approved by the Regional Ethical Committee and conformed to the principles of the Declaration of Helsinki. Clinical characteristics of the cohort are described in Supplementary table 1. All patients were examined with a preoperative carotid ultrasound and were clinically assessed by a neurologist. All patients included were considered eligible for carotid endarterectomy, according to clinical decision in a multidisciplinary meeting with neurologists, angiologist, radiologists, clinical physiologists and vascular surgeons. Patients were considered symptomatic if they suffered from amaurosis fugax, transient ischemic attack (TIA) or ischemic stroke within one month prior to surgery. The indications for carotid endarterectomy were 1) ipsilateral symptoms and stenosis > 70% or 2) asymptomatic patient with > 80% stenosis as assessed by duplex ultrasound as previously described.(1) No power calculation was possible due to the design of the study. The inclusion was done consecutively and no exclusion criteria were used (except the ability to provide informed consent). No outliers were removed from the statistical analysis. The clinical data in this study was collected by research nurses at the time of inclusion and in vitro data was compiled by the technical and research staff. The research subjects were only patients being operated for a carotid stenosis, with the criteria for operation specified above. Replicates were used as much as possible considering the limited and valuable human material studied. The in vitro experiments consist of both analysis of levels of proteins and mRNA in plaque tissue homogenates, as well as cell culture experiments with THP-1 macrophages (all described in detail in their respective methods section). The data was processed randomly and blinded for the technical staff.

*Sample Preparation*

Carotid plaques were commonly removed using an eversion technique (obtaining two larger pieces of plaque: carotid communis and interna) and snap-frozen in liquid nitrogen in the operation theatre immediately after surgical removal. The plaques were wet weighed on dry ice before dissection into the different fragments used for each part of the study (RNA sequencing, histology, plaque tissue homogenate). From the most stenotic part of the plaque a 1 mm thick section was taken for histology and embedded in optimal cutting medium (OCT, Sakura Finetek Europe BV, Japan). An adjacent section of 1 mm was kept for RNA sequencing. The rest of the plaque was homogenized in a standardised way, described earlier. (2)

*Histology, immunohistochemistry and immunoflourescence*

To stain for the three TGF-β isoforms the frozen tissue was cut in an approximately 2 mm slice, which was fixated in 4% buffered formaldehyde solution (Histolab Products AB) overnight. The tissue was then dehydrated in increasing alcohol concentrations, cleared in Xylen and finally embedded in Histowax (Histolab Products AB). Mouse monoclonal antibodies were used to stain for TGF-β1 (Abcam, Cambridge, UK, ab27969, 0.06 µg/ml) and TGF-β2 (Abcam, Cambridge, UK, ab36495, 0.25 µg/ml) overnight at 4^o^C. A mouse monoclonal isotype control antibody was used as a control (Abcam, Cambridge, UK, ab81032, at corresponding concentration). A rabbit polyclonal antibody was used to stain for TGF-β3 (Abcam, Cambridge, UK, ab15537; 0,293 mg/ml) together with a rabbit polyclonal isotype control antibody (Abcam, Cambridge, UK, ab37415, 5 mg/ml). To detect staining a MACH3 probe and horseradish peroxidase (HRP) polymer (Biocare Medical, Pacheco, CA, USA) was used.

Positive immunoreactivity was visualized using 3,3'-diaminobenzidine (DAB; Vector Laboratories Inc, Burlingame, CA, USA). Sections were counterstained with Mayer’s Hematoxylin (Histolab, Gothenburg, Sweden). Stained sections were scanned and digitalized using Aperio ScanScope digital slide scanner (Aperio Technologies, Inc, Vista, CA) and immunoreactivity was quantified using the imaging software program BioPix iQ version 2.3.1 (Biopix Ab, Gothenburg, Sweden).

Immunoflourescent stainings were performed on formalin fixed decalcified (EDTA 14%, pH7) paraffin embedded tissue section (5 µm). Sections were treated with HIER Citrate buffer pH6 and BSA (10%) was used for blocking. A monoclonal mouse antibody was used stain for TGFB1; 0,24µg/ml (Abcam, ab27969). A polyclonal rabbit antibody was used to stain for TGFB2; 1µg/ml (Proteintech, 28426-1-AP). A rabbit polyclonal antibody was used to stain for TGFB3; 4 ug/ml (Abcam, 15537). A monoclonal rabbit antibody to Alpha Actin Smooth Muscle Actin; 0,5µg/ml (Abcam, ab124964) and a mouse monoclonal antibody to Alpha Actin Smooth Muscle Actin, 0,1µg/ml (Dako, M0851) were used to stain for smooth muscle alpha-actin. A rabbit monoclonal antibody to CD68; 0,24µg/ml (Cell Signaling #76437) and mouse monoclonal antibody to CD68; 1µg/ml (Dako, M0814) were used to stain for CD68. Polyclonal Donkey anti- Mouse Alexa Fluor 555; 2µg/ml (Abcam, ab150106) and polyclonal Goat anti-Rabbit Alexa Fluor 488; 2µg/ml (Abcam, ab150074) antibodies were used as secondary antibodies. All slides were mounted using Vectashield Mounting medium for IF with DAPI (Vector Laboratories, H1200).

*Biochemical assessments of plaque glycosaminoglycans, collagen and elastin levels*

The ECM components glycosaminoglycans (GAG), collagen and elastin were analysed in plaque homogenate with colometric assays as previously described.(3) In brief, GAGs were measured using Blyscan proteoglycan and glycosaminoglycan assay (Biocolor, Carrickfergus, UK), collagen was measured using the Sircol collagen assay (Biocolor Carrickfergus, UK) and elastin was measured using the Fastin elastin assay (Biocolor, Carrickfergus, UK). Values were normalized to plaque wet weight

*Plaque levels of cytokines, matrix metalloproteinases, tissue inhibitors of matrix metalloproteinases and oxidized LDL*

Cytokines were measured in plaque homogenate supernatants using Luminex (Human Cytokine/Chemokine Immunoassay, Millipore Corporation, MA, USA), analysed with Luminex 100 IS 2.3 (Austin, TX, USA) as previously described, or a proximity extension assay (Olink, Uppsala), as previously described.(4,5) All cytokines were normalized to plaque wet weight. The proximity extension assay analysis was performed by a preprocessing normalization procedure using Olink Wizard for GenEx (Multid Analyses, Sweden) and all data is presented as arbitrary units.

Matrix metalloproteinases (MMPs) -1, -2, -3, -9, and -10 were analysed with Mesocale human MMP ultra-sensitive kit (Mesoscale, Gaithersburg, MD, USA) and tissue inhibitors of MMPs (TIMPs 1 and 2) were analysed using MILLIPLEX MAP Human TIMP Magnetic Bead Panel (Milliplex, MA, USA) in plaque homogenate supernatants as previously described.(6) All analyses were performed according to the manufacturer's instructions and results were normalized to plaque wet weight.

Plaque homogenate supernatant levels of oxLDL were assessed using ELISA (Mercodia, Uppsala, Sweden). The analysis was performed in accordance to the manufacturer´s instructions. Supernatants were obtained by centrifugation of plaque homogenate at 12000 g.”

*LPS stimulation of THP-1 cells*

Human blood monocytes (THP-1, 88081201, Sigma, Saint Louis, USA) were cultured in RPMI-1640 medium (11875093, Thermo Fisher Scientific, Waltham, USA) supplemented with 10 % FBS (10270106, Thermo Fisher Scientific, Waltham, USA) and 50 U/mL penicillin-streptomycin. Prior to the experiment, cells were treated with Phorbol 12-Myristate 13-Acetate (PMA, cat #78139 Sigma, Saint Louis, USA) 100ng/mL for 24 hours, and then washed 3 times with PBS and medium changed every 2 days for 6 days. The resulting adherent”M0” macrophages were then treated with 5 ng/mL TGF-β1, -β2, -β3 (T2815, Sigma, Saint Louis, USA) for 48 hour and successively with 100 ng/mL LPS (L2630, Sigma, Saint Louis, USA) for 15 hours to polarise towards a pro-inflammatory macrophage phenotype.

*Oxidized LDL stimulation of THP-1 cells*

Human THP1 monocytes were cultured in RPMI 1640 medium supplemented with 10% fetal bovine serum, 100 U/mL penicillin, 100 𝜇g/mL streptomycin, and 0.05 mmol/L 2-mercaptoethanol. The cells were seeded in 12-well plates (2× 105 cells/well) and preincubated with 100 ng/mL phorbol 12-myristate 13- acetate (PMA, Sigma) for 24 hours to differentiate into macrophages as experimental cells. The 3 days rested THP1 macrophages were preincubated with 10 ng/mL of TGF-β1, -β2, or -β3 and subsequently treated with oxLDL (20µg/ml, Thermo Fisher Scientific) for 24 hours. Thereafter the cells were harvested and total RNA was extracted using RNeasy Mini Kit (Qiagen). Total RNA (1𝜇g) was reverse-transcribed using high-capacity RNA-to-cDNA kit (Thermo Fisher, Göteborg, Sweden) according to the manufacturer’s instructions. Gene expression of MCP1 and MMP9 was examined by quantitative real-time PCR on a QuantStudio 7 Flex instrument (Applied Biosystems/Thermo Fisher) using Taqman Fast Advanced master mix and appropriate Taqman probes. Relative gene expression was calculated with QuantStudio Software v1.1 (Thermo Fisher) using the ΔΔCt method and normalized to GAPDH expression as endogenous control (n=5 for all groups). For comparisons between different groups, gene expression levels were expressed as fold change expression compared to control samples (untreated THP1 macrophages).

*In vitro RNA analyses*

For the in vitro experiments, total RNA was extracted using the Trizol method and reverse transcribed using the high capacity RNA-to-cDNA kit (Thermo Fisher, Göteborg, Sweden). Gene expression was analyzed by quantitative real-time PCR on a QuantStudio 7 Flex instrument (Applied Biosystems/Thermo Fisher) using Taqman Fast Advanced master mix and the following Taqman probes (Thermo Fisher): Hs00234140_m1 (CCL2/MCP-1), Hs00957562_m1 (MMP9), Hs02786624_g1 (GAPDH), Mm00441242_m1 (Ccl2/MCP-1), Mm00442991_m1 (Mmp9), Mm99999915_g1 (Gapdh). Relative gene expression was calculated with QuantStudio Software v1.1 (Thermo Fisher) using the ΔΔCt method and normalization to GAPDH expression as endogenous control (n=3 for all groups). The qPCR gene expression data is normalized to expression of endogenous control and for comparison of groups, gene expression levels are expressed as fold change expression compared to controls.

*RNA Sequencing*

Expression of the TGF-β isoforms and their receptor genes were evaluated from global transcriptome RNAseq data collected from 69 plaques from the same cohort. RNA was prepared from standard total RNA extraction with Trizol cleared of Ribosomal RNA using Ribo-Zero™ Magnetic Kit from (Epicentre). Strand specific RNAseq libraries were prepared with ScriptSeq™ v2 RNA-Seq Library v2 Preparation Kit (Epicentre).

Paired-end sequencing libraries were generated and sequenced using high-output kit version 2, HiSeq2000 platform, Illumina, USA. Quality and adapter trimming were performed using TrimGalore! (Version 0.3.7, Babraham Bioinformatics, Cambridge, UK) with the default parameters (Quality Phred score cutoff is 20, minimum retained length per read is 20 bp).

Transcript expression for transcripts annotated in Gencode v.27 was quantified using Salmon in mapping-based mode, and, subsequently, gene-level counts were summarized using tximport. Gene counts were normalized between samples using trimmed mean of M-values (TMM) by edgeR, giving gene expressions as log2-transformed counts per million (CPM) after voom transformation. Batch effects of flow cell were then adjusted by an empirical Bayes method.

After quality control 60 plaques were used in correlation and differentially gene expression analyses. Spearman’s correlation between genes were performed. A linear regression was conducted to examine differentially expressed genes comparing patients with (symptomatic) or without symptomatic carotid disease (asymptomatic). P-values were adjusted using the Benjamini & Hochberg (BH) method.

*Microarray plaque analysis in the BiKE cohort*

For the microarray analysis, carotid plaques from the Biobank of Karolinska carotid Endarterectomies (BiKE; Karolinska Institute, Stockholm, Sweden) project were obtained with participants´ informed written consent, per the Declaration of Helsinki and approved by the Ethical Committee of Stockholm. A total of 127 atherosclerotic plaque tissue samples were collected from patients who underwent carotid endarterectomy (CEA) at Karolinska University Hospital, Stockholm, Sweden. After being rinsed, the plaques were frozen instantly on dry ice for subsequent isolation of RNA. Total RNA was extracted with a RNeasy Mini kit according to the instructions of the manufacturer. The tissue samples were assessed and samples with low-quality RNA and low concentrations were excluded from the study. RNA samples were hybridized and scanned at the Karolinska Institute Affymetrix core facility using Affymetrix HG-U133 plus 2.0 arrays.

*Plaque levels of endoglin*

Endoglin, the TGF-β2 co-receptor, was measured in plaque tissue homogenate supernatants after centrifugation for 5 min at 5000 RCF in 4°C, using the sandwich Endoglin Elisa kit (antibodies-online, Aachen, Germany) according to the manufacturer’s instructions and was normalized to plaque wet weight.

**References**

1. Hansen F, Bergqvist D, Lindblad B, Lindh M, Mätzsch T, Länne T. Accuracy of Duplex Sonography before Carotid Endarterectomy - A Comparison with Angiography. *European Journal of Vascular and Endovascular Surgery*. 1996; 12:331-6
2. A. Edsfeldt, I. Goncalves, H. Grufman, M. Nitulescu, P. Duner, E. Bengtsson, I. G. Mollet, A. Persson, M. Nilsson, M. Orho-Melander, O. Melander, H. Bjorkbacka, J. Nilsson, Impaired fibrous repair: a possible contributor to atherosclerotic plaque vulnerability in patients with type II diabetes. *Arterioscler Thromb Vasc Biol. 2014;*34:2143-2150.

I. Goncalves, J. Moses, N. Dias, L. M. Pedro, J. Fernandes e Fernandes, J. Nilsson, M. P. Ares, Changes related to age and cerebrovascular symptoms in the extracellular matrix of human carotid plaques. *Stroke. 2003;* 34:616-622.

Goncalves, A. Edsfeldt, N. Y. Ko, H. Grufman, K. Berg, H. Bjorkbacka, M. Nitulescu, A. Persson, M. Nilsson, C. Prehn, J. Adamski, J. Nilsson, Evidence Supporting a Key Role of Lp-PLA2-Generated Lysophosphatidylcholine in Human Atherosclerotic Plaque Inflammation. *Arterioscler Thromb Vasc Biol*. 2012;32:1505.

Rattik S, Wigren M, Bjorkbacka H, Fredrikson GN, Hedblad B, Siegbahn A et al. High plasma levels of heparin-binding epidermal growth factor are associated with a more stable plaque phenotype and reduced incidence of coronary events. *Arterioscler Thromb Vasc Biol.* 2015;35:222-8.

1. A. Edsfeldt, I. Goncalves, H. Grufman, M. Nitulescu, P. Duner, E. Bengtsson, I. G. Mollet, A. Persson, M. Nilsson, M. Orho-Melander, O. Melander, H. Bjorkbacka, J. Nilsson, Impaired fibrous repair: a possible contributor to atherosclerotic plaque vulnerability in patients with type II diabetes. *Arterioscler Thromb Vasc Biol*. 2014;34:2143-2150.

**Supplementary Table 1.** Clinical characteristics of the 223 patients (127 symptomatic and 96 asymptomatic) included in the study from Carotid Plaque Imaging Project (CPIP) cohort.

|  | **All**  **(n=223)** | | **Symptomatic (n=107)** | **Asymptomatic**  **(=96)** | **p-value** |
| --- | --- | --- | --- | --- | --- |
| Age (years) | 70 (64-76) | | 74 (65-79) | 68 (64-71) | 0.000002 |
| Males n (%) | 151 (67.7) | | 73 (68) | 66 (68.8) | 0.739 |
| Smoking n (%)* | 68 (30.5) | | 26 (24.3) | 36 (37.5) | 0.28 |
| Hypertension n (%) ** | 166 (74.4) | | 78 (81.3) | 74 (69.2) | 0.2 |
| Lipid-lowering treatment n (%) | 191 (85.7) | | 90 (84.1) | 86 (89.6) | 0.20 |
| Degree of stenosis (%) | 90 (80-95) | | 90 (75-95) | 90 (85-95) | 0.334 |
| Hs-CRP (mg/L) | 3.7 (1.9-6.3) | | 3.8 (2.0-6.7) | 3.6 (1.5-5.9) | 0.252 |
| HbA1c (mmol/mol) | 43.4 (38-54.7) | | 44.0 (39-58) | 42 (38-50) | 0.251 |
| ***Fasting lipoproteins (mmol/L)*** | |  |  |  |  |
| Total Cholesterol | | 4.3 (3.5-5.1) | 4.3 (3.6-5.1) | 4.3 (3.5-5.1) | 0.760 |
| LDL | | 2.5 (1.9-3.2) | 2.5 (2.0-3.3) | 2.25 (1.8-3.1) | 0.211 |
| HDL | | 1.1 (0.9-1.3) | 1.1 (0.9-1.2) | 1.1 (0.9-1.4) | 0.975 |
| Triglycerides | | 1.3 (0.9-1.8) | 1.3 (1.0-1.8) | 1.3 (0.9-1.9) | 0.493 |

HDL, high density lipoproteins; LDL, Low density lipoproteins; Hs-CRP, high sensitive c-reactive protein. Categorical variables are expressed in number (percentages) and continuous variables as median (interquartile range). Categorical variables were analysed with chi-square test and continuous variables with Mann-Whitney’s test. All patients with symptoms >1month prior to surgery were excluded in comparison between symptomatic and asymptomatic patients. * Active smoking for at least 6 months before surgery. ** Systolic blood pressure > 140 mmHg or treated for hypertension

**Supplementary Table 2.** Summary of cerebro- and cardiovascular events and deaths in the 1st-3rd tertile compared to patients in the 4th quartile of TGF-β2.

|  | **1^st^-3^rd^ quartile** | **4^th^ quartile** |
| --- | --- | --- |
| *Cerebrovascular event, n* | 14 | 4 |
| *Cardiovascular event, n* | 16 | 2 |
| *Cerebrovascular death, n* | 8 | 0 |
| *Cardiovascular death, n* | 13 | 1 |

**Supplementary Table 3.** Levels of TGF-β1, -β2 and -β3 in human carotid plaque homogenates, symptomatic and asymptomatic patients presented separately

|  | All  (n=223) | Symptoms<1month (n=107) | Asymptomatic  (n=96) |
| --- | --- | --- | --- |
| TGF-β1 | 182 (97-347) | 175 (80-305) | 162 (104-312) |
| TGF-β2 | 1261 (802-2252) | 1047 (710-1573) | 1436 (846-2759) |
| TGF-β3 | 62 (34-109) | 54 (34-90) | 63 (30-120) |

Values are presented as median (interquartile range) and the values are in pg/g wet weight plaque. Plaques associated with symptoms >1month prior to surgery were removed from the comparison between symptomatic and asymptomatic plaques (n=20).

**Supplementary Table 4.** VIP scores for all variables with significant impact (VIP>1) on classification of symptomatic and symptomatic patients. TGF-β2, Transforming Growth Factor Beta 2; TIMP1, Tissue Inhibitor of Metalloproteinases 1; MMP3, Matrix Metalloproteinase 3; MMP9, Matrix Metalloproteinase 9; IL6, Interleukin 6; MCP-1, Monocyte Chemoattractant Protein-1;MMP2, Matrix Metalloproteinase 2; MIP-1b, Macrophage Inflammatory Protein 1-Beta; MMP1, Matrix Metalloproteinase 1; MMP10, Matrix Metalloproteinase 10; TIMP2, Tissue Inhibitor Of Metalloproteinases 2; TGF-β3, Transforming Growth Factor Beta 3; TGF-β1, Transforming Growth Factor Beta 1; HDL, High-density lipoprotein; LDL, Low-density lipoprotein; CRP, C-Reactive Protein

**Supplementary Table 5.** Spearman correlation between the three isoforms of TGF-β (-β1, -β2 and -β3) and the time between symptoms and surgery (n=127).

|  | **TGF-β1** | | **TGF-β2** | | **TGF-β3** | |
| --- | --- | --- | --- | --- | --- | --- |
|  | **r** | **p** | **r** | **p** | **r** | **p** |
| Time between symptoms and surgery (days) | 0.256 | 0.004 | 0.208 | 0.019 | 0.290 | <0.001 |

TGF, Transforming Growth Factor

**Supplementary Table 6.** Correlation analyses revealed significant correlations between free protein levels of TGF-β2 and the downstream target proteins MMP-3 (n=208) and PAR-1 (n=186). Spearman correlation test was used.

|  | TGF-β1 | TGF-β2 | TGF-β3 |
| --- | --- | --- | --- |
| *PAR-1* | r=0.22** | r=0.662*** | r=0.405*** |
| *MMP-3* | r=0.1 | r=0.17* | r=0.13 |

TGF, Transforming Growth Factor. PAR-1, protease activated receptor-1. MMP-3, matrix metalloproteinase-3. *p<0.05, ***p<0.0001.

**Supplementary Table 7.** Uni- and multivariate Cox proportional hazard regression model exploring associations between TGFB2 and future cardiovascular events.

|  | Hazard ratio (95%CI) | | | | Hazard ratio per 1SD increase of TGFB2 (95% CI) | P |
| --- | --- | --- | --- | --- | --- | --- |
|  | Q1 | Q2 | Q3 | Q4 |  |  |
| Model 1 | 1 (ref) | 0.81 (0.48-1.39) | 0.68 (0.39-1.21) | 0.38 (0.20-0.71) | 0.67 (0.51-0.88) | 0.004 |
| Model 2 | 1 (ref) | 0.83 (0.48-1.45) | 0.75 (0.42-1.35) | 0.43 (0.22-0.84) | 0.71 (0.54-0.93) | 0.01 |
| Model 3 | 1 (ref) | 0.80 (0.45-1.42) | 0.61 (0.32-1.15) | 0.41 (0.20-0.83) | 0.69 (0.52-0.93) | 0.02 |

Model 1, unadjusted. Model 2, adjusted for age and sex. Model 3 adjusted for age, sex, smoking, total cholesterol, high density lipoprotein and hypertension. CI: confidence interval; Q: quartile; SD: standard deviation; P: p-value; ref: reference

**Supplementary Figures**

**Supplementary** **Figure 1.** Human plaque tissue homogenates were spiked with recombinant TGF-β1/2/3 and the free forms of TGF-β1/2/3 were measured using Milliplex MAP TGF-β1/2/3 magnetic bead kit to confirm the accuracy of method used to measure plaque levels of the three isoforms. Approximately 60-70% of the three isoforms were recovered and no significant differences between the three isoforms were detected.

**Supplementary Figure 2.** Carotid plaque mRNA levels of TGF-β2 was validated in the BiKE cohort (Karolinska Institute, Stockholm, Sweden). **A)** TGF-β2 mRNA levels were significantly lower in symptomatic compared to asymptomatic plaques. Bars represent the mean and whiskers represent the standard deviation. Blue bars indicate asymptomatic and red bars indicate symptomatic plaque levels (n=127). *,p≤0.05 with Students T-test**.**

A)

**

B)

**

**Supplementary Figure 3. Immunohistochemistry showed that asymptomatic carotid plaques have a more stable plaque phenotype compared to symptomatic plaques.** A) Plaque areas of lipids (Oil red O), glycophorin A (intra plaque haemorrhage) and CD68 (macrophages) were significantly greater in symptomatic plaques compared to asymptomatic. B) A calculated vulnerability index (a ratio between plaque area stained positive for Oil red O, glycophorin A and CD68 divided by plaque area stained positive for alpha-actin and collagen) was also significantly lower in asymptomatic compared to symptomatic plaques. Mann Whitney U test was used for the statistical comparison.

1. B)

**Supplementary** **Figure 4**. Plaque levels of endoglin, measured in carotid plaque tissue homogenates, showed to trend towards significant differences comparing symptomatic and asymptomatic plaques. Mann Whitney U test was used for the statistical comparison.


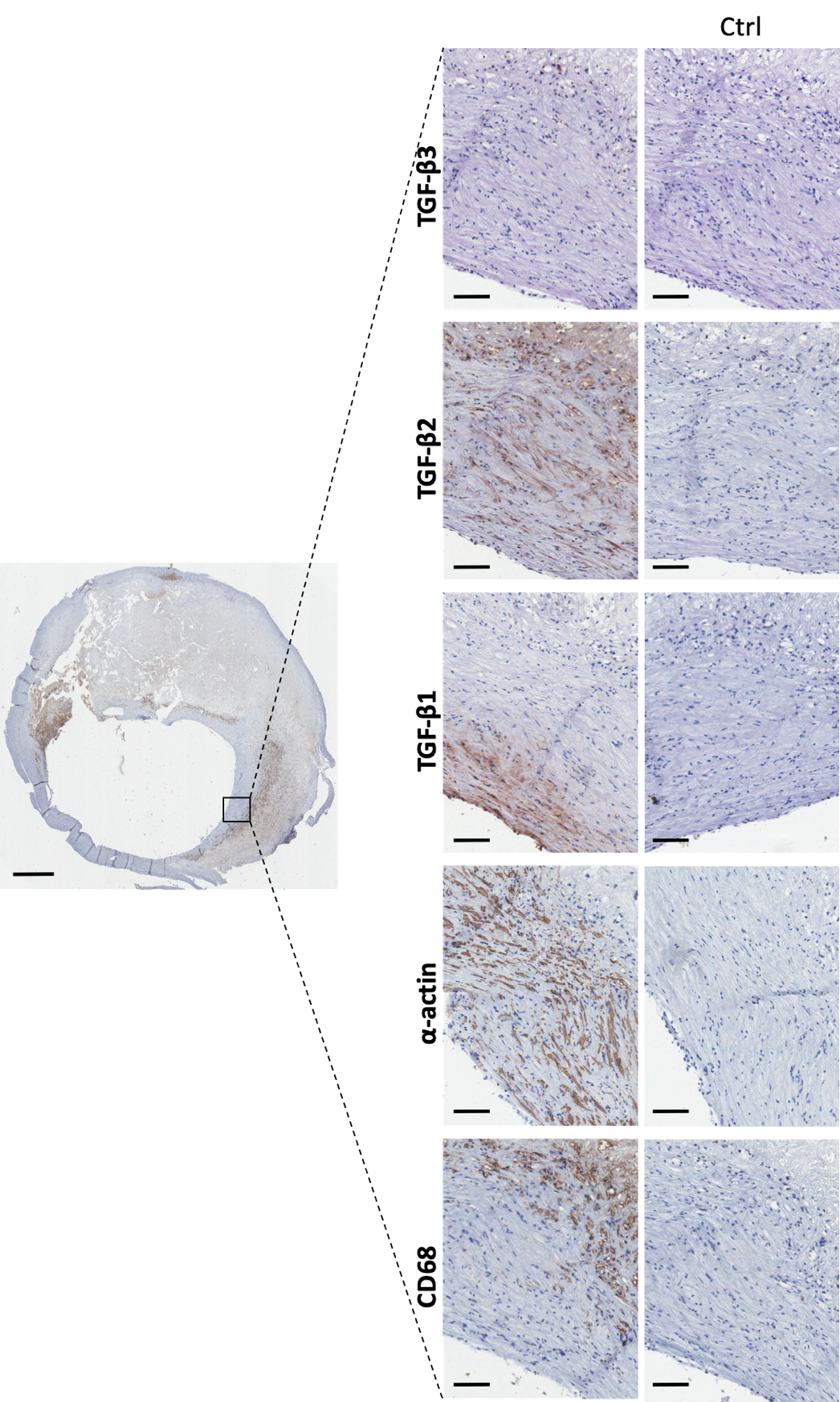


**B)**


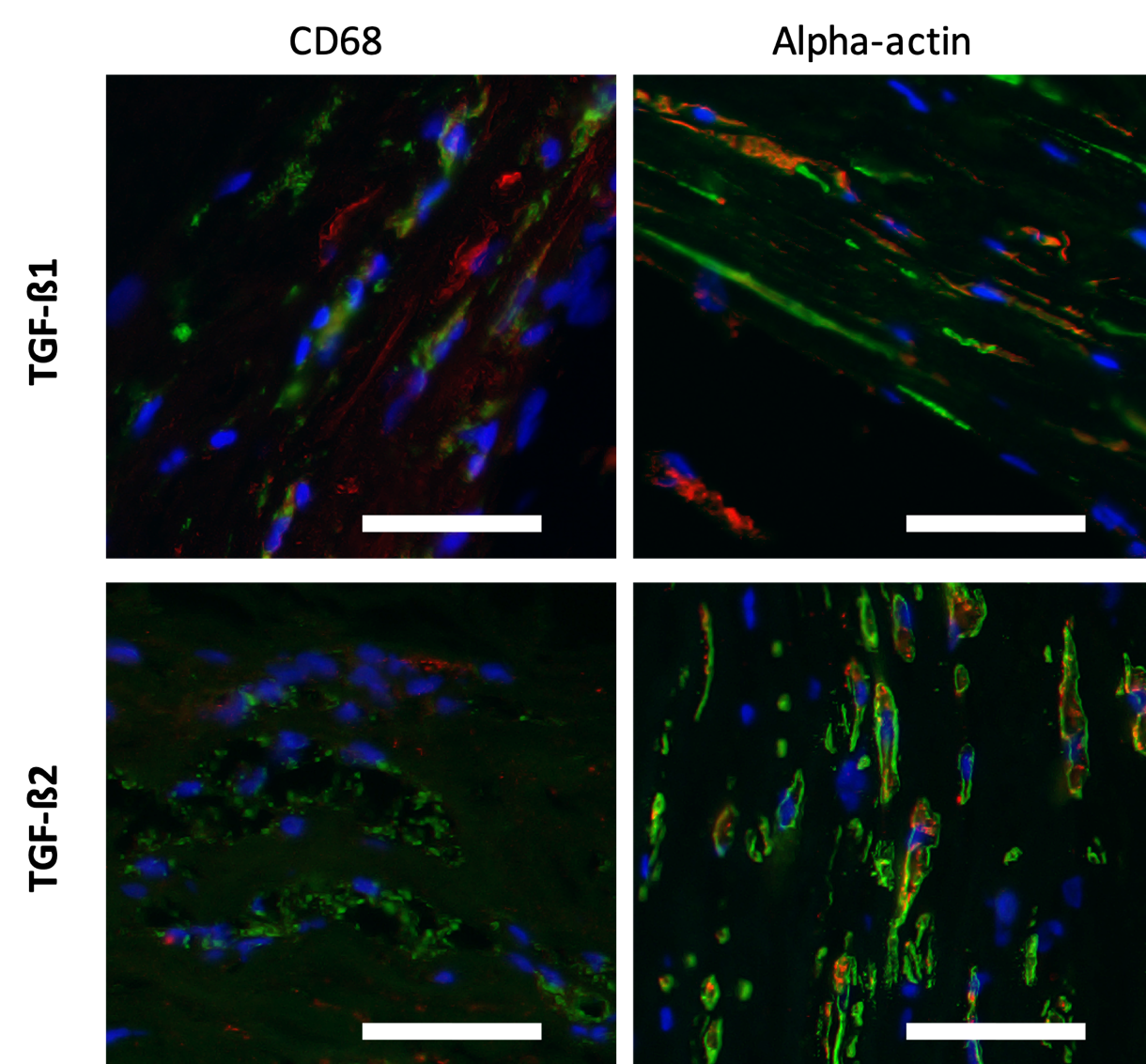


**Supplementary** **Figure 5**. **A)** Immunohistochemistry (n=5) and **B)** immunofluorescence showed co-localization of TGF-β2 and alpha-smooth muscle actin in human carotid atherosclerotic plaques. CD68, cluster of differentiation 68. CTL, control. TGF-β1 and -β2 in red. CD68 and alpha-smooth muscle actin in green. DAPI in blue. Scale bars in A) 1mm in overview image (left panel; CD68 stained in brown) and 100um in magnified areas (middle and right panels) and in B) 50um.

**Supplementary** **Figure 6.**  Plaque protein levels of TGF-β signalling target proteins, matrix metalloproteinase 3 and protease-activated receptor-1, were significantly higher in asymptomatic plaques compared to symptomatic plaques. Mann Whitney U tests were used. n=91 vs 100 for MMP3 and n=89 vs 96 for PAR-1.

1. B)

**Supplementary Figure 7.** TGF-ß1, TGF-ß2 and TGF-ß3 reduced the oxidized LDL induced gene expression of **A)** MCP-1 whereas only TGF-ß1, and TGF-ß3 reduced **B)** MMP9 gene expression in PMA matured THP-1 cells. One-way ANOVA was used for statistical analyses. n=5.*, p<0,05. Ns, non significant.


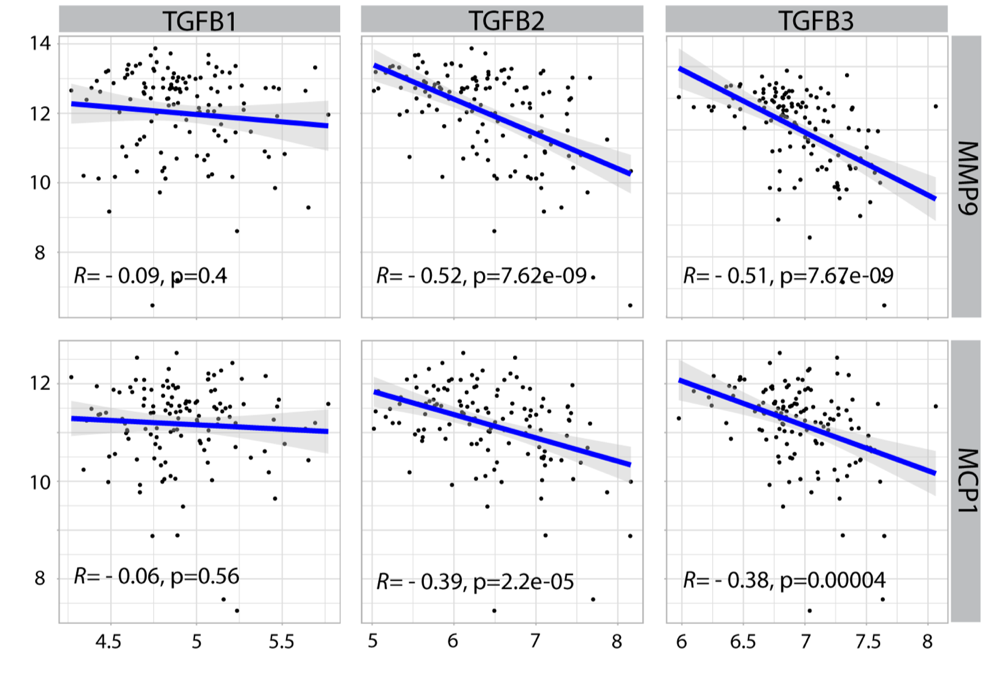


**Supplementary Figure 8.** Carotid plaque mRNA levels of TGF-β2 in the BiKE cohort (Karolinska Institute, Stockholm, Sweden) showed strong inverse correlations to MMP-9 and MCP-1 mRNA levels. Pearson’s correlation test was used (n=127).

A)


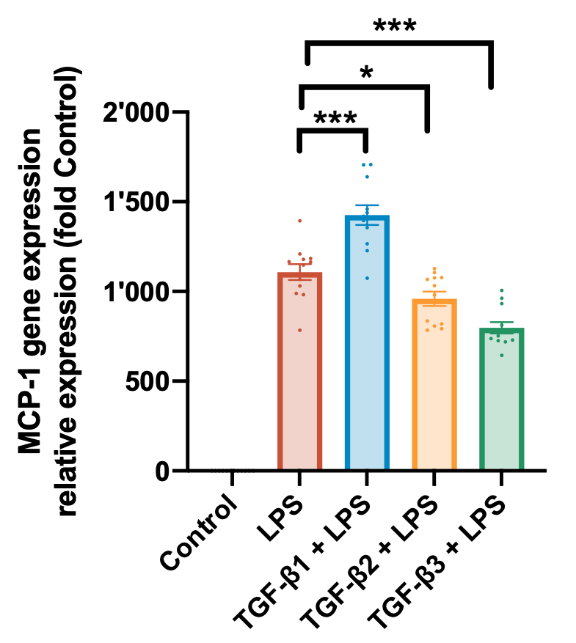

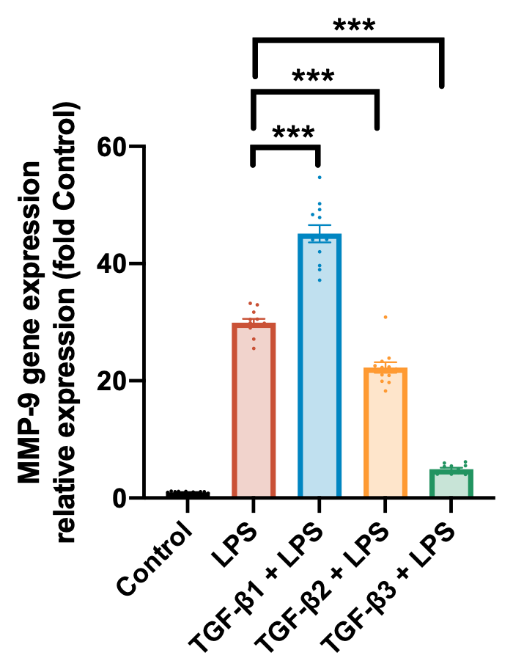


**Supplementary** **figure 9.** TGF-β2 and TGF-β3 pre-stimuli reduced MCP-1and MMP-9 gene expression in RAW264.7 cells upon LPS stimuli. Kruskal Wallis and Mann Whitney U test were used. n=12 for each group. ***p<0.001, *p<0.05.

A) B)

**Supplementary figure 10** **A)** Distribution of Z-scores from the cox-regression analysis comparing 4^th^ quartile vs 1^st^ – 3^rd^ quartiles of TGF- β2 plaque protein levels and the risk for future cardiovascular events. **B)** Distribution of Z-scores from cox-regression using TGF-β2 as continuous variable. Blue vertical line represents Z-score from the cox regression using the unshuffled TGF-β2.
